# A Method for Estimating Gaseous Emissions in Multistory Car Parks, Based on Scenarios and Particularities: MB-UPT Case Study

**DOI:** 10.3390/toxics14100865

**Published:** 2026-09-29

**Authors:** Adriana Tokar, Daniel Bisorca, Danut Tokar, Daniel Muntean, Andreea-Miruna Tokar

**Affiliations:** 1 Department of Civil Engineering and Building Services Engineering, Civil Engineering Faculty, University Politehnica Timișoara, 300006 Timisoara, Romania; adriana.tokar@upt.ro (A.T.); danut.tokar@upt.ro (D.T.); daniel-beniamin.muntean@upt.ro (D.M.); 2 Faculty of Medicine and Pharmacy, University of Oradea, 410087 Oradea, Romania; tokar.andreeamiruna@student.uoradea.ro

**Keywords:** estimating method, air pollutant, vehicle, polluting gaseous emissions, vehicle emission factors, multistory car parks, operating mode

## Abstract

The need to build multistory parking in congested areas of cities presents new challenges regarding the estimation of the impact of these investments on the environment and neighboring buildings. The authors synthesize the data available in the official EU documentation and in the scientific literature and propose a method for calculating polluting gaseous emissions generated in multistory car parks. For the case study targeting the MB-UPT car park, a scheme with functional blocks was created, with elements that quantitatively and qualitatively influence the polluting gaseous emissions (CO, CO_2_, NMVOC, NOx, NH_3_) generated by the operation of the car park. The study considers a fleet of motor vehicles with characteristics like those of the motor vehicles in the car park in Timiș County. The calculation method was applied considering three operating scenarios with high-level emissions, estimated to be encountered in real operation. Considering the operating modes of the motor vehicles, emission factors and correction coefficients were chosen for the highest possible degree of confidence in the emission estimates. The results are presented graphically and in tabular form. From the analysis of the results, for each pollutant species analyzed, the relevant critical factor was determined in the context of the three scenarios. The method proposed by the authors allows for the quantitative determination of polluting gaseous emissions and easy identification of episodes and zones with high emissions.

## 1. Introduction

The concentration of the population in urban areas and the improvement of living standards can be considered causes of the increase in the number of vehicles in cities, along with the increasing need for parking space. Most of the problems generated by the lack of parking spaces are reflected in traffic congestion, traffic rule violations, accidents, etc. [1,2,3]. Furthermore, areas prone to traffic congestion generate high levels of polluting gaseous emissions that pose a significant health risk to pedestrians and occupants of nearby buildings [4,5,6,7]. One of the urban planning problems in Romania is the lack of parking spaces (1.2 million public parking spaces) for registered vehicles (approximately 7 million) [8,9]. Due to the fact that the existing infrastructure could not keep up with the increase in the number of vehicles, the problem of insufficient parking spaces has persisted for some time. In most large cities in the EU, it appears necessary to ensure the availability of organized parking spaces (in the vicinity of public transport stations, shopping centers, educational campuses, public institutions, etc.), in accordance with sustainable urban mobility measures [10,11].

Regarding these aspects, the scientific literature offers studies that address the problem of transport infrastructure development and insufficient car parking spaces in EU countries and that conclude that major investments, which are related to urban mobility solutions, are needed [1,12,13,14,15,16]. A recent approach concerns parking spaces for autonomous vehicles and the impact that this type of vehicle has on urban area management [17,18,19,20].

Given the lack of available spaces for the construction of parking structures, a challenge of urban development is the incorporation of parking spaces into multifunctional structures, such as the one addressed in this article (the MB-UPT car park).

The concentration of a high number of vehicles generates challenges related to noxious emissions inside and near parking structures (aboveground/underground). If these car parks are proposed to be built near residential/public buildings, a prior assessment of the polluting gaseous emissions during the operation of this type of construction is required. The official EU literature (developed by the European Environment Agency) [21,22,23] provides data and theoretical mathematical models for estimating specific gaseous emissions from road vehicles, applicable to various conditions (road sectors, vehicle fleet structure, operating regimes, weather conditions, etc.). From the analysis of this data, it was concluded that the official EU literature does not provide sufficient information regarding estimating emission factors to cover all situations likely to be encountered in operating vehicles in parking spaces, namely ramp travel and driving with a cold engine.

Also, in the scientific literature, there are applied studies developed by various teams of researchers [4,5,24,25,26,27,28,29] that present their own models and estimates of emission factors, specifically of correction coefficients, but these studies are not connected with the official data presented/recommended in the official EU literature. At the EU level, it is required that impact studies be developed based on emission factors from the official EU literature. However, there are situations that are not covered by emission factor values and/or calculation relationships for polluting gaseous emissions.

For this reason, the study developed by the authors proposes an integrated/cumulative approach to the data and methods offered by the EU official specialized literature [21,22,23] and the scientific specialized literature [4,5,24,25,26,27,28,29], and presents a calculation method adapted to car-park-type structures where most of the situations are likely to be encountered in real operation.

Correct estimation, specifically the application of corrections for emission factors and the calculation of gaseous emissions with a high degree of confidence, is essential for carrying out an impact study that reflects as accurately as possible a real mode of operation of car parks. The polluting gaseous emissions thus calculated will constitute qualitative input data (similar to real emissions) that can be used to model the impact of the use of parking spaces, both on the occupants of the building in question and neighboring buildings, and on the surrounding environment.

Problems related to polluting gaseous emissions from car parks come from both loading/emptying flows correlated with the degree of use and stationary emissions resulting from the evaporation of fuels from the tanks of parked motor vehicles.

Subsequently, after an investment is made, we aim to validate:

- The proposed methodology, through measurements of local polluting gaseous emissions inside the car parks for motor vehicles;
- Numerical modeling of the impact of the objective on air quality to determine the concentration of polluting gaseous emissions in the area in which the emission receptors are located, in both the various spaces of the MB-UPT and in proximity to neighboring buildings, through immission measurements.

Even though Timisoara is among the cities in Romania with the most parking spaces, it still faces the need for their availability in the most crowded areas of the city. One of these areas is the campus of the University Politehnica Timisoara (UPT), where, during the academic year, parking spaces are insufficient.

**Figure 1:**
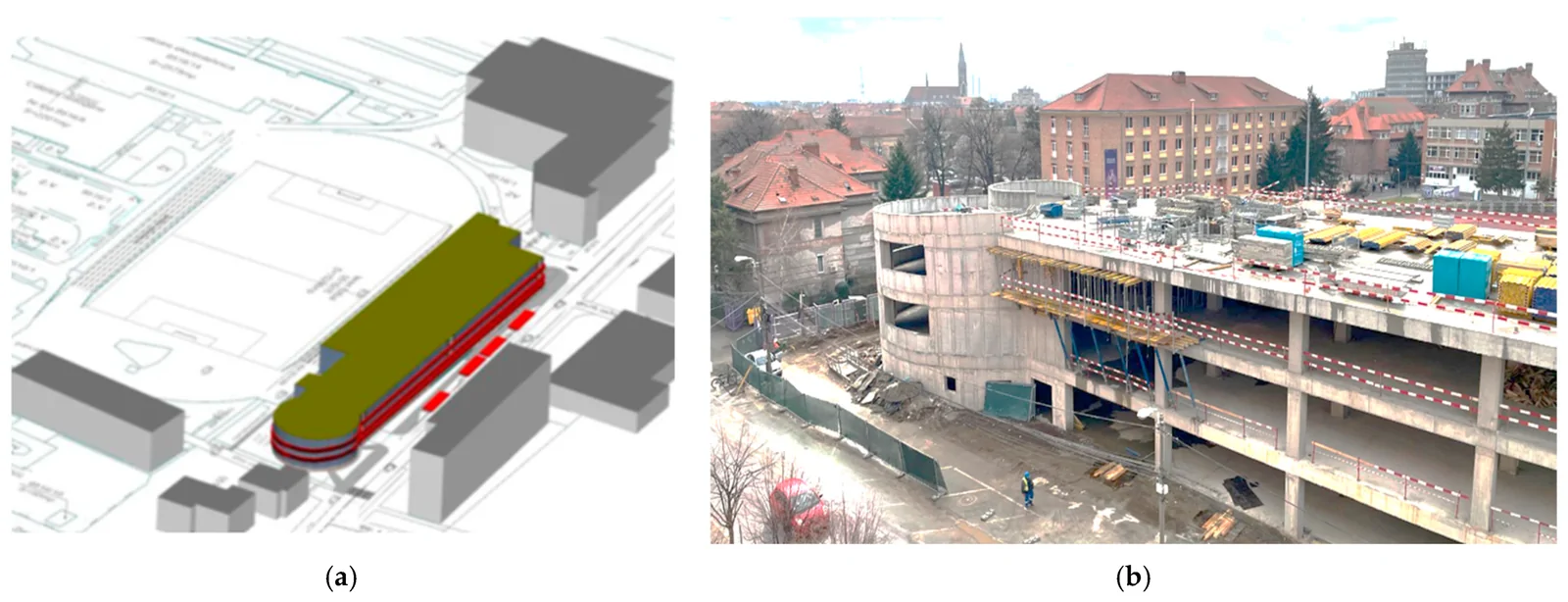
Proposed multifunctional car park—MB-UPT: (**a**) sketch—MB-UPT car park integration; (**b**) photo—MB-UPT car park construction site.

Currently, identifying additional parking spaces is a challenge, but a solution was found involving the decommissioning of the old construction of the UPT sports base stadium with the grandstand destination and two mini-football-fields in the immediate vicinity. Practically, the solution consists of the development of a multifunctional construction (Figure 1) with the role of an aboveground car park on three levels that encompass the stadium stands, two sports fields and spaces related to sports activities. The development of the construction will largely solve the problem of parking spaces in this area of the city of Timisoara.

Practically, the following distinct functions are proposed for the multifunctional building (MB-UPT): spaces for coach parking on the ground floor (four spaces), spaces for car parking on the ground floor, first floor and second floor (60 spaces/level), spaces for servicing sports activities related to the adjacent football field (a fitness room and a conference room), bleachers for spectators (1484 seats), spaces for two sports fields with their own locker rooms (six locker rooms for athletes and referees), café with terrace, technical and building maintenance spaces and two elevators for vertical circulation (for easier access/for vertical circulation).

Pollution sources are located at different heights as follows:

- Ground floor of the multifunctional building (H_level_ = 0 m) with a built area of 3778.10 sq m, including:
  - Parking area for four coaches;
  - Controlled-access vehicle entrance area;
  - Parking area: 56 spaces;
  - Vehicle parking area for people with locomotor disabilities: four spaces;
  - Vehicle exit area;
  - Circular ramp for vehicle access to the first floor.
- The first floor of the multifunctional building (H_level_ = 3.75 m) with a built area of 3956.90 sq m and a usable area of 665.25 sq m, comprising:
  - Parking area: 56 spaces;
  - Parking area for vehicles for people with locomotor disabilities: four spaces;
  - Circular ramp for vehicle access to the second floor.
- The second floor of the multifunctional building (H_level_ = 7.5 m) with a built area of 2864.10 sq m and a usable area of 222.55 sq m, comprising:
  - Parking area: 58 spaces;
  - Parking area for vehicles for people with locomotor disabilities: two spaces;
  - Circular ramp for vehicle access to the building’s terrace.

The inclusion of an aboveground car park in a multifunctional structure requires studies on the impact of the exploitation of parking spaces, both on its own spaces and on the existing buildings in the immediate vicinity. The construction that includes the car park has three levels (ground floor, first and second floors and terrace respectively), is oriented along the North–South axis and has a total of 180 parking spaces.

**Figure 2:**
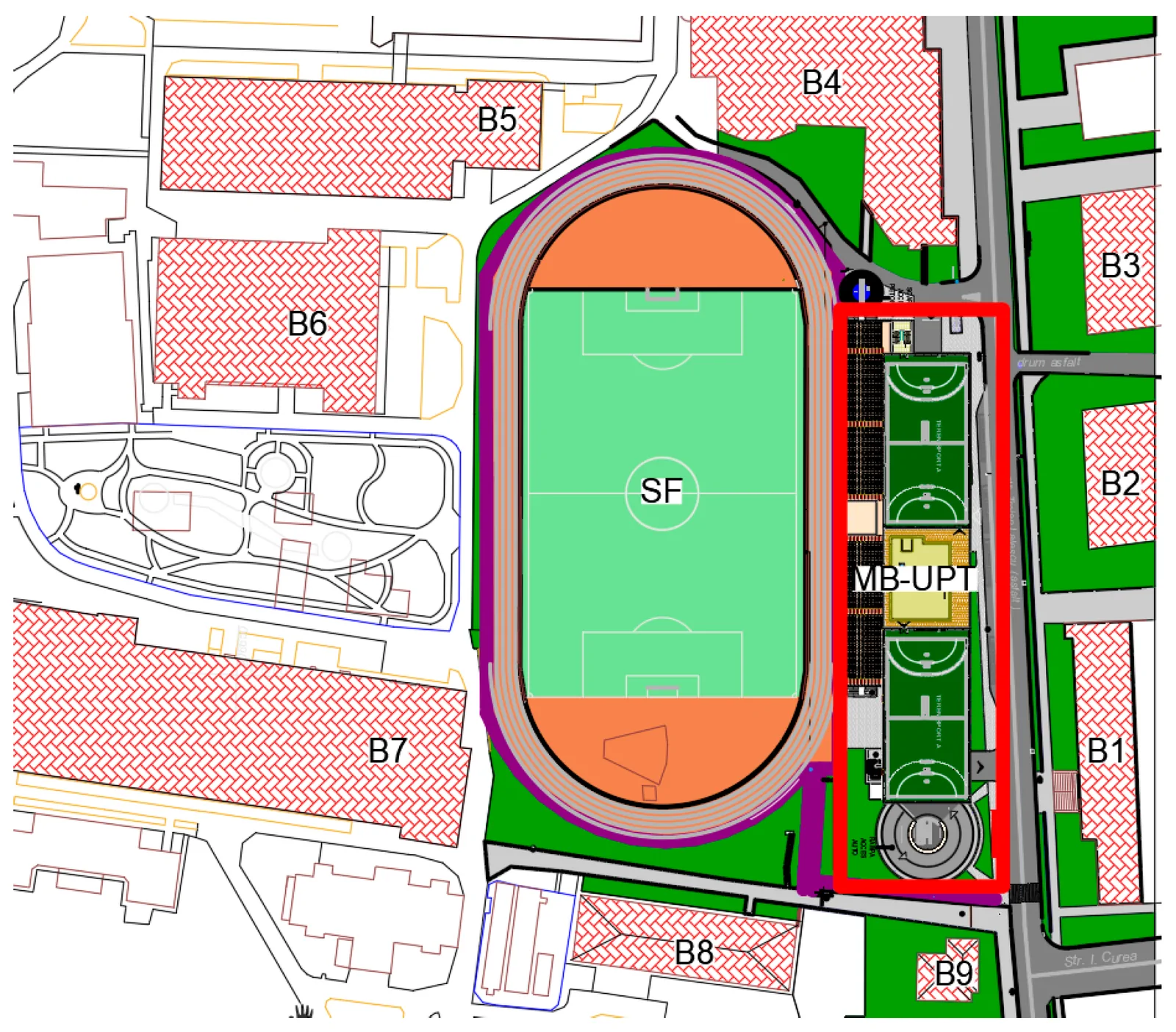
Top view of the area of interest. Existing situation.

The land plot on which the building is being constructed is located between the “Ştiința” football field of the University Politehnica of Timisoara (UPT) and Traian Lalescu Street, with an estimated size of L = 150.00 m and l_avrage_ = 35.00 m. The construction is located in the vicinity of the following buildings: Faculty of Civil Engineering (B1) with a height regime of SB + GF + 5F + A (opposite to the east, at a distance of 26.8 m), INCERC building (B2) with a height regime of its ground floor (opposite to the east, at a distance of 39.1 m), construction of the sports hall of the West University of Timisoara (WUT) (B3) with a height regime of GF + 1F (opposite to the east, at a distance of 29.38 m), UPT Library (B4) with a height regime of GF + 3F (to the north, at a distance of 27.25 m), the Faculty of Electrical Engineering (building C) (B5) (northwest, at a distance of 85.14 m), the Faculty of Mechanics (Horology building) (B6) (to the west, at a distance of 119.78 m), the Laboratory of Faculty of Mechanics (B7) with a height regime of GF + 1F (to the west, at a distance of 94.58 m), student dormitory 2MV (B8) with a height regime of GF + 4F (southwest, at a distance of 26.60 m), residential building with a height regime of GF + 2F (B9) (to the south, at a distance of 13.18 m) and the UPT Sports Field (SF) (football) (to the west, at a distance of 8.72 m); the buildings are represented in Figure 2, on which the area intended for the investment has been highlighted with a red outline.

The placement of the spectator bleacher ensures good visibility of the sports activities taking place on the sports field. For motor vehicle access, a controlled-access entrance was provided in building B4, and the exit for motor vehicles is directly on Traian Lalescu Street, localized near the vertical traffic ramp (Figure 1).

The article proposes the development of a method for calculating polluting gaseous emissions generated in multistory car parks, applied to a case study (the MB-UPT car park). This study complements the literature by determining hourly gaseous emissions taking into account scenarios and particularities related to the academic calendar, the vehicle fleet, the operating regime of the motor vehicle engines (cold engine or hot engine), the mode of driving of the vehicles (horizontal plan driving, uphill or downhill on a ramp) and evaporative emissions from the tanks of parked vehicles.

## 2. Materials and Methods

**Figure 3:**
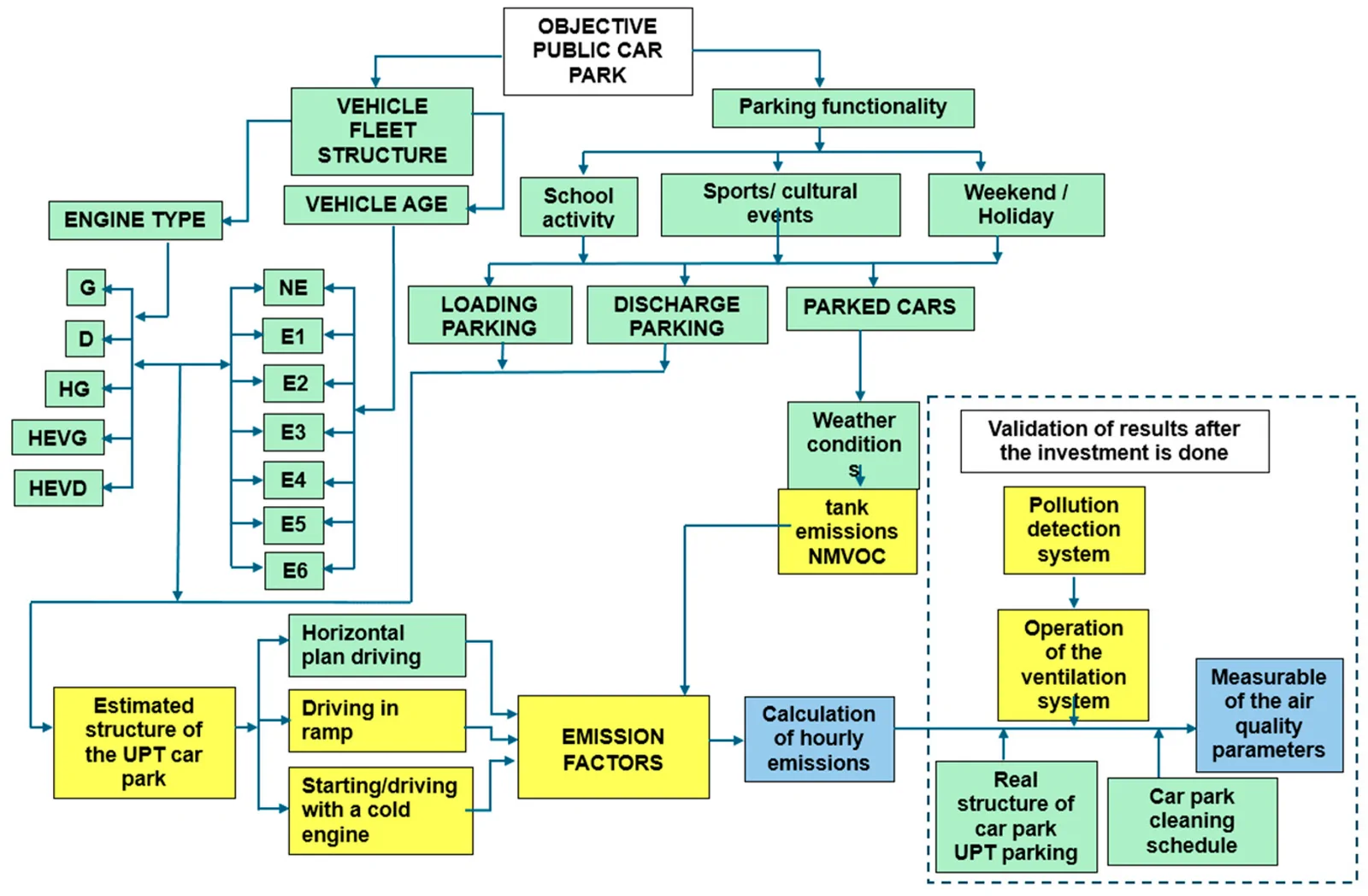
The proposed connection and interaction scheme for establishing the input data necessary for the development of the calculation method. G—Gasoline, D—diesel, HG—hybrid gasoline, HEVG—plug-in hybrid electric vehicle (gasoline), HEVD—plug-in hybrid electric vehicle (diesel).

In the first stage, the scheme with functional blocks, connection lines, inputs and outputs was created, whose components quantitatively and qualitatively influence the polluting emissions generated by the operation of the MB-UPT car park (Figure 3).

The scheme with functional blocks has been color-coded to indicate the availability of data in the official EU and/or scientific literature and/or official EU and/or scientific databases, as follows:

- Green—available data;
- Yellow—estimated by extrapolation;
- Blue—calculated based on available equations.

The functionality of the parking lot was analyzed from the point of view of average hourly use, specific to the MB-UPT car park (for example, the structure of the 2024–2025 academic year was considered):

- School activity period (30 September 2024–20 December 2024; 8 January 2025–16 February 2025; 24 Febaruary 2025–17 April 2025; 28 April 2025–6 July 2025);
- Weekends with/without sports/cultural events (occasionally);
- Holidays (21 December 2024–7 January 2025; 18 April 2025; 27 April 2025; 7 July 2025–29 September 2025).

For each of these periods, in accordance with the teaching activities program, the situations that can generate maximum hourly gaseous emissions of pollutants inside the car park were analyzed and identified, over significant intervals:

○ ***A period of school activity:***
  - 7.00–8.00—Occupancy accumulation phase;
  - 8.00–14.00—Stationary phase;
  - 14.00–15.00—Emptying phase;
  - 15.00–16.00—Occupancy accumulation phase;
  - 16.00–20.00—Stationary phase;
  - 20.00–22.00—Emptying phase.
○ ***Weekends and periods with organized sports/cultural events:***
  - With sports/cultural events:
    ▪ Loading 2 h before the event;
    ▪ Parked—during the event (from 3 to 8 h);
    ▪ Emptying 60 min after the event.
  - Without sports/cultural events:
    ▪ A 10% loading factor was considered for practicing individual sports activities.
○ ***School holidays***—A 30% loading factor was considered for the university’s administrative staff and for practicing individual sports activities.

For the vehicles using the car park, specific emission and correction factors were identified from the official EU literature [21,22,23], for the following types of sources, norms and operating regimes:

- Types of engine (gasoline, diesel, hybrid gasoline, plug-in hybrid electric vehicle (gasoline), plug-in hybrid electric vehicle (diesel));
- European emission standards (non-Euro, Euro 1–Euro 6);
- VOC emissions;
- Engine operating mode (cold/hot);
- Vehicle driving mode (ramp/horizontal plane).

Subsequently, the data available in the specialized literature (the scientific literature and the official EU literature) was identified and presented for each component of the connection and interaction scheme, namely:

- Analysis of data available in the specialized literature regarding emission factors by fuel type, pollution standards, operating regimes and vehicles driving modes, and fugitive VOC emissions from fuel tanks;
- Data customization by correlating hourly load levels with parking duration and outside air temperature;
- Data collection on the dimension and the structure of local car fleet from Timisoara.

### 2.1. Analysis of Data Available in the Specialized Literature Regarding Emission Factors

In the specialized literature, the European Environment Agency (EEA)’s *Air Pollutant Emission Inventory Guidebook* provides information and informative methodologies on the values of emission factors specific to road vehicles [21]. The guide specifies, for motor vehicles, the types of polluting gaseous emissions: CO, CO_2_, NMVOC, NOx, and NH_3_ [21] which are presented in Table 1. The emission factors’ values for all types of pollutants and respectively the fuel consumption Fuel_cons_ in g/kg were considered for the medium vehicle category for the following engine types: petrol, diesel, hybrid petrol and plug-in hybrid electric vehicle (petrol), and respectively plug-in hybrid electric vehicle (diesel) for the large category related to the engine type.

A separate category of factors considered in this study refers to emission factors related to evaporative emissions specific to gasoline-fueled motor vehicles. Evaporative emissions for diesel motor vehicles were neglected due to the presence of heavy hydrocarbons and the low vapor pressure of diesel fuel. When assessing evaporative emissions, stationary losses are considered (evaporation of gasoline from a tank due to changes in temperature and pressure), a method which takes into account an average emission factor established for each motor vehicle category [21]. For the case study considering gasoline PC motor vehicles, the emission factor values are presented in Table 2. These emission factors vary significantly depending on the temperature range during the day, being higher for higher temperatures. The specialized literature [22] provides approximate values for different daily emission factors to cover temperature ranges from 20 to 35 °C, 10 to 25 °C, 0 to 15 °C and −5 to 10 °C respectively. For the case study, the emission factor values were calculated by interpolation in intervals of 5 degrees Celsius and were expressed in g/vehicle/h. The calculation of polluting gaseous emissions was carried out taking into account loading grade of the car park MB-UPT and the hourly temperature evolution inside the car park.

### 2.2. Data on the Correlation of Hourly Load Levels with Parking Duration, Outside Air Temperature, and Roadway Slope

The slope of the roadway has a considerable effect on polluting gaseous emissions because it affects the engine’s load regime [24].

From the point of view of the engine’s operating regime (hot or cold), polluting gaseous emissions were considered for the unfavorable scenarios described previously.

The polluting gaseous emissions’ values from the specialized literature were selected for the following vehicle driving modes:

- Horizontal plan driving: 0% slope;
- Ramp travel (uphill/downhill): 10% slope:
  ○ Entry in parking—hot engine;
  ○ Exit from parking—cold engine start.

For horizontal plan driving, the emission factors are those presented in Table 1, and for traffic on a 10-degree ramp during uphill (hot engine) and downhill (cold engine), the correction factors in Table 3 were considered [29].

The cold start emission factors depending on the indoor temperature of the parking spaces are presented in Table 4, and the correction factors depending on the parking duration (f_ds_) are summarized in Table 5 [29]. The multiplication factors for the distance driven with a cold engine inside the car park (f_d_) are presented in Table 6 and for the age of the motor vehicle fleet (f_fa_) are summarized in Table 7 [29].

Since in the official literature [29] there are no multiplication coefficients for the CO_2_ and NH_3_ species related to cold engine operation (at start-up), the authors used values obtained by interpolation of data available in the scientific literature [30,31], namely: multiplication coefficient of 1.34 for CO_2_ [30] and 1.7 for NH_3_ [31] respectively, values which are representative for an average temperature of 0 °C.

### 2.3. The Motor Vehicle Fleet Structure from Timis

**Figure 4:**
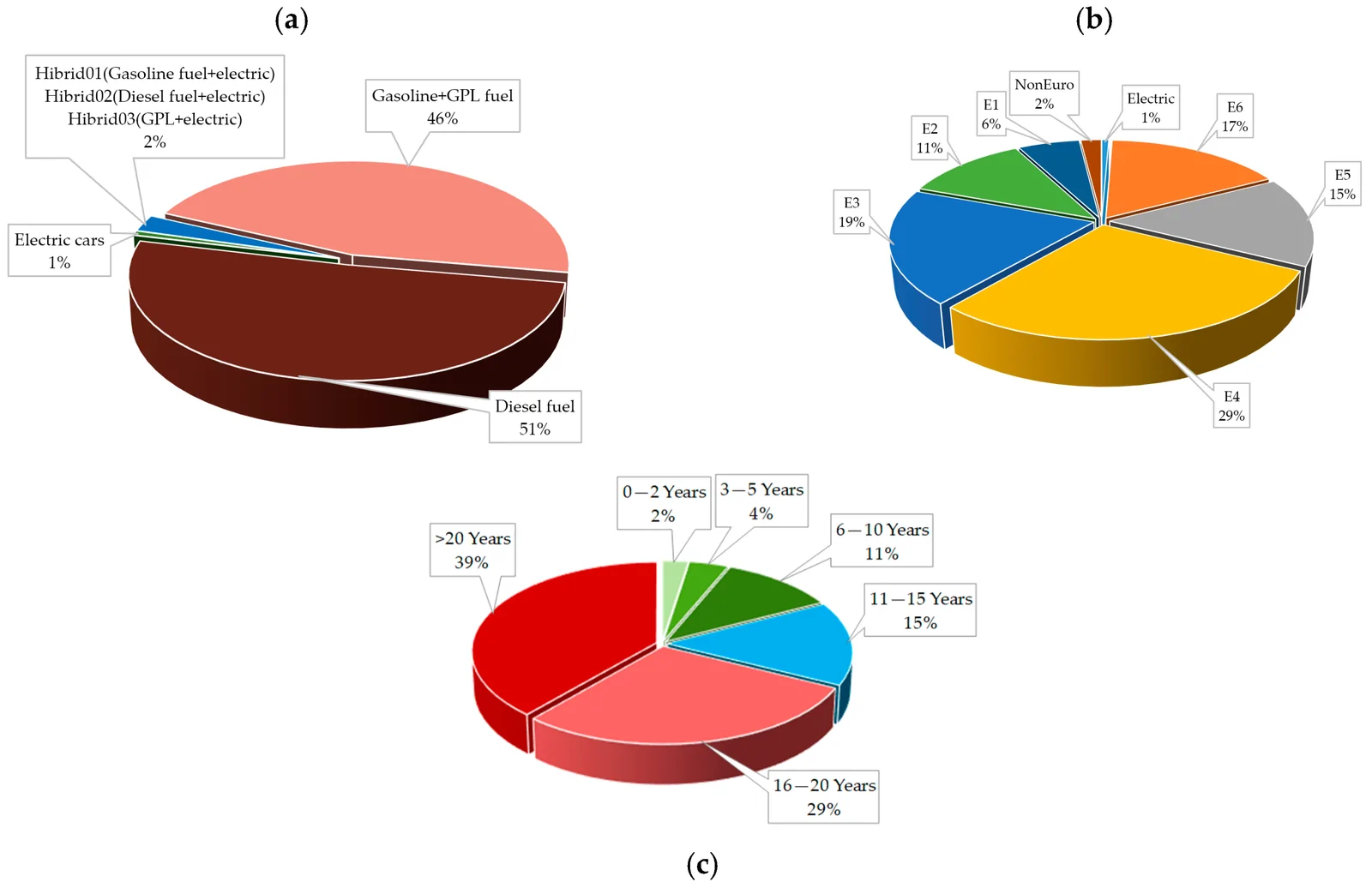
Structure of the motor vehicle fleet in Timiș County. (**a**) Function of motorization; (**b**) function of European emission standards; (**c**) function of age.

The compositions of the motor vehicle fleet in Timis County are presented in Figure 4, as follows: for vehicles according to engine type (Figure 4a), European emissions standards (Figure 4b), and age (Figure 4c) [32].

### 2.4. Calculation Method

The emission factors’ values and the structure of the motor vehicle fleet from Timis County in 2024 (data available at the time of the study) were used in the formulation of the equation that formed the basis for determining the hourly emissions.

The calculation of air-polluting gaseous emissions from the MB-UPT car park activity was carried out by combining the information available in the specialized literature with estimates and interpolations presented in the method developed by the authors.

The polluting gaseous emissions from parking lots can be divided into:

- Emissions from thermal engines (exhaust gases) from the vehicle driving mode (entry—hot engine, exit—cold engine): CO, NOx, benzene, etc.
- Polluting gaseous emissions from the process of gasoline evaporation: NMVOC.

The polluting gaseous emissions calculated for the case study concerned the following gaseous pollutants: CO, CO_2_, NMVOC, NO_x_ and NH_3_. Combustion emissions (from exhaust gases) are calculated taking into account a series of factors that influence the value of these emissions. Thus, in addition to data on the driving distance of vehicles from the entry point to the parking space and from the parking space to the exit, a series of correction factors are also used. The correction factors considered in the equations depend on the parking duration, emissions from cold engine start, the number of entry–exits, the number and category of motor vehicles (pollution class), and the type of fuel used by the motor vehicles (petrol or diesel).

To estimate the EF_eh_vc_level_ emissions from horizontal plan driving of vehicles which are looking for a parking space (hot engine), for each building level the general Relationship (1) was used:

(1) $${EF}_{eh\_vc\_level}=\sum_{i=1}^{n}{(N}_{m\_he\_i}{\cdot REF}_{eh\_vc\_i}\cdot d_{med\_level}),$$

where EF_eh_vc_level_ refers to the hourly emissions from horizontal plan driving while looking for a parking space, in g/h; i refers to motor vehicle categories and European emission standards (Table 1); n refers to estimated total number of motor vehicle categories and European emission standards for each level (dimensionless); N_m_he_i_ refers to hourly number of motor vehicles driven with a hot engine in category i within the defined spatial boundary, h^−1^; REF_eh_vc_i_ refers to the reference emission factor per motor vehicle category (Table 1) specific to the type of pollutants, calculated in g/km; d_med_level_ refers to the average distance driven by a motor vehicle in category i, inside the car park on the current level, in km.

The estimation of EF_ec_vc_ emissions from the driving of motor vehicles in the process of leaving the parking space (cold engine), starting and driving with a cold engine on a horizontal plane on each level was made using Equation (2):

(2) $${EF}_{ec\_vc\_level}=\sum_{j=1}^{n}{[V}_{m\_ce\_j}\cdot min\left(d_{j}\cdot f_{d};1\right)\cdot f_{cs}\cdot f_{ds}\cdot f_{fa}],$$

where EF_ec_vc_level_ refers to the hourly emissions from cold starting and driving of motor vehicles on the respective level, in g/h; j refers to number of motor vehicles per level (dimensionless); n refers to total number of vehicles driving in the car park on each level (dimensionless); V_m_ce_j_ refers to vehicles starting and moving with a cold engine within the defined spatial boundary (dimensionless); f_cs_ refers to emissions from cold start depending on the outside temperature (Table 4), in g/vehicle_start_; f_ds_ refers to the correction factor for cold-starting vehicles, depending on the duration of parking (Table 5) (dimensionless); min(d∙f_d_;1) refers to the reduction factor dependent on the distance driven by the motor vehicle with a cold engine (dimensionless); d_j_ refers to the distance driven by the current vehicle until leaving the car park, in km; f_d_ refers to the correction factor for the distance driven with a cold engine inside the car park (Table 6) (dimensionless); f_fa_ refers to the correction factor depending on the age of the motor vehicle fleet (Table 7) (dimensionless).

The factors from Equation (2) are universally valid for all types of engines according to the specialized literature [29]. Considering that 95% of the analyzed motor vehicle fleet is older than 5 years (Figure 4b), the influence of the age of the motor vehicles was neglected in the calculation relation (2) because the specialized literature indicates a value of 1 for the age coefficient of the vehicles until 2020 [29].

Regarding the method of estimating hourly evaporative emissions from the tanks of parked motor vehicles on each level, the general Relation (3) was used, considering that data on the types of engines of the motor vehicles and the daily temperatures inside the car park are available.

(3) $$E_{VOC\_level}=N_{pi}\cdot {EF}_{VOC,i},$$

where E_VOC_level_ = the hourly emissions of NMVOC, în g/h; N_pi_ = the hourly number of parked vehicles in category i on the current level (dimensionless); EF_VOC,i_ = the emission factor of VOC for vehicle category i (Table 2); j is in g/vehicle/h; i = the vehicle category (passenger cars, light-duty vehicles and two-wheel vehicles, i.e., mopeds and motorcycles).

No HDVs and buses are considered, as the share of gasoline-fuelled motor vehicles in these categories is too low to be considered for evaporative emissions.

Based on the calculation relationships in the specialized literature [29], the engine emissions during running of hot engines were calculated for motor vehicle movement on a ramp, using the calculation Relationship (4), a relationship that is valid for all vehicle categories.

(4) $$EF_{vc\_ramp}=\sum_{i=1}^{n}{(N}_{m\_he\_i}{\cdot REF}_{eh\_vc\_i}\cdot d_{ramp\_level}\cdot f_{r}),$$

where EF_vc_ramp_ refers to the hourly emissions from the ramp travel of motor vehicles looking for a parking space, in g/h; i refers to number of vehicle categories and European emission standards (Table 1); N_m_he_i_ refers to number of vehicles moving with a hot engine in category *i* within the defined spatial boundary (dimensionless); REF_eh_vc_i_ refers to the reference emission factor per vehicle category (Table 1) specific to the type of pollutants, calculated in g/km; d_ramp_level_ refers to the distance driven by a motor vehicle on the ramps between the three levels of the building, in km; f_r_ refers to correction factors for driving on ramp (Table 3) (dimensionless).

The emission factor EF_vc_ramp_ was calculated only for the uphill ramp, considering that during downhill the vehicles have insignificant emissions due to the use of engine braking.

The total emission on each level (TE_v_level_) can be estimated using Relationship (5), being expressed in g/h.

(5) $${TE}_{V\_level}={EF}_{eh\_vc\_level}+{EF}_{ec\_vc\_level}+E_{VOC\_level}$$

The total emissions calculated on the ramp between two levels (TE_v_ramp_) can be estimated using Relation (6), being expressed in g/h.

(6) $${TE}_{V\_ramp}=EF_{vc\_ramp}$$

The calculation of emission factors considered a fleet of motor vehicles classified according to:

- The type of fuel used by the motor vehicles (petrol or diesel) and the classification of the vehicles using the European emission standards (Table 1);
- The operating regime of the motor vehicle engines (cold engine or hot engine);
- The mode of driving of the vehicles (horizontal plan driving, uphill or downhill on a ramp).

## 3. Results and Discussion

**Figure 5:**
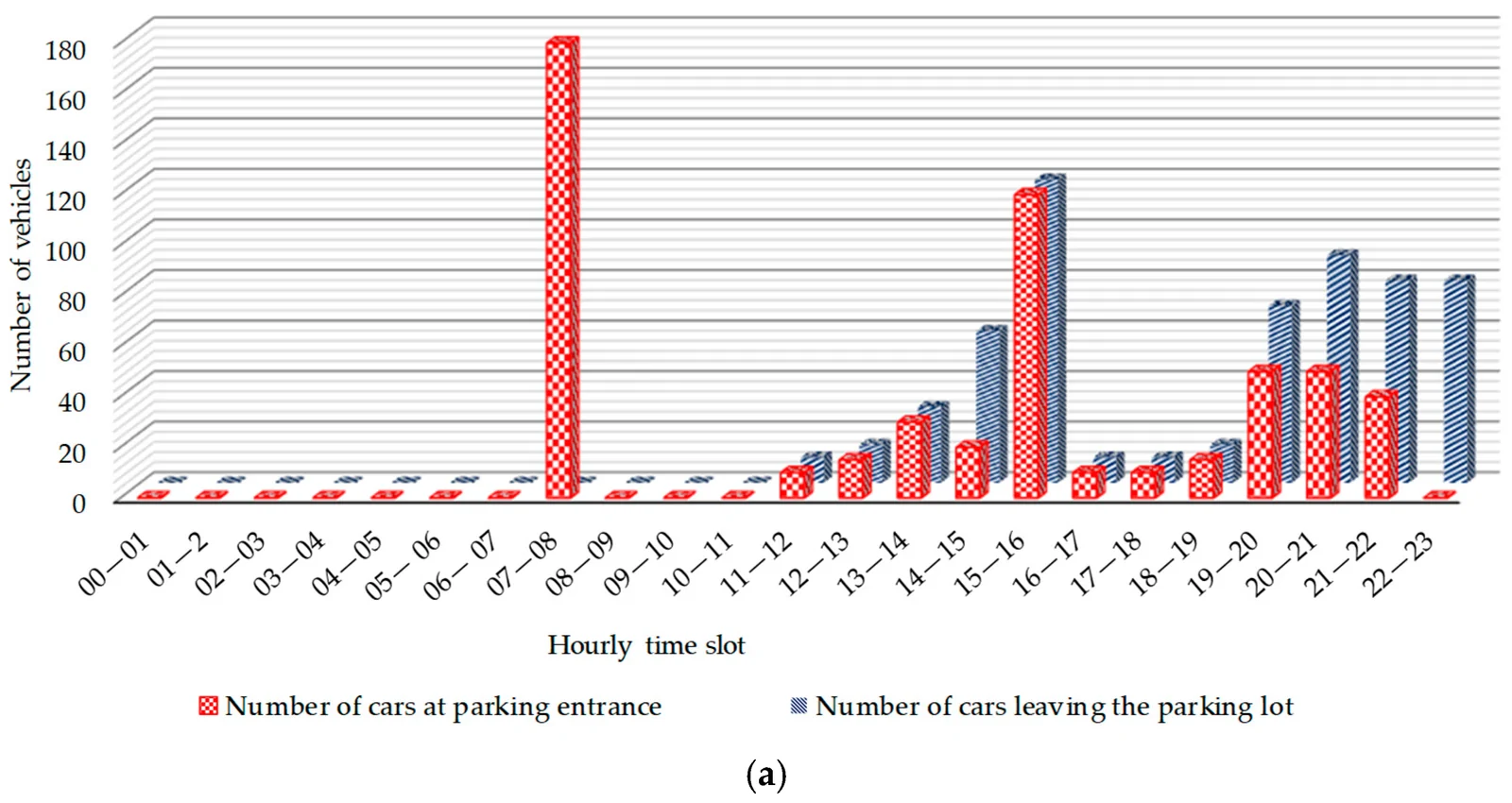

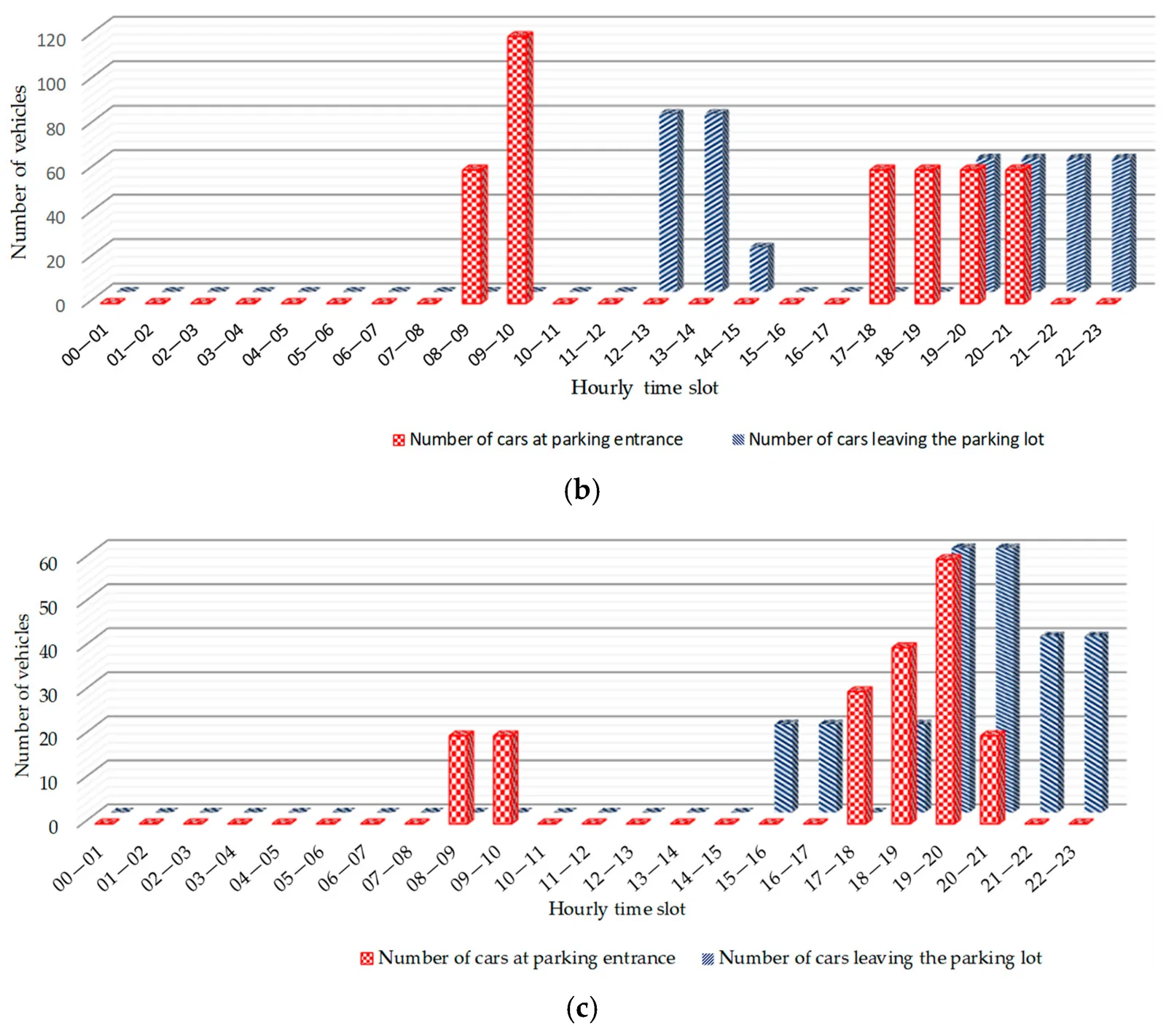
Estimated hourly evolution of the number of vehicles entering or leaving the parking lot. (**a**) School activity period; (**b**) period with sports/cultural events; (**c**) holiday period.

For all the atmospheric air pollution sources presented above, the periods with characteristic maximum emissions were extracted, resulting from the operating period of the objective. The authors identified three scenarios for the MB-UPT parking lot correlating with the structure of the UPT academic year. The analyzed scenarios and the obtained results are presented below. Figure 5 shows the expected (estimated) hourly evolution of the number of vehicles entering or leaving the parking lot, related to the characteristic periods (school activity, sports/cultural events, holiday).

**Figure 6:**
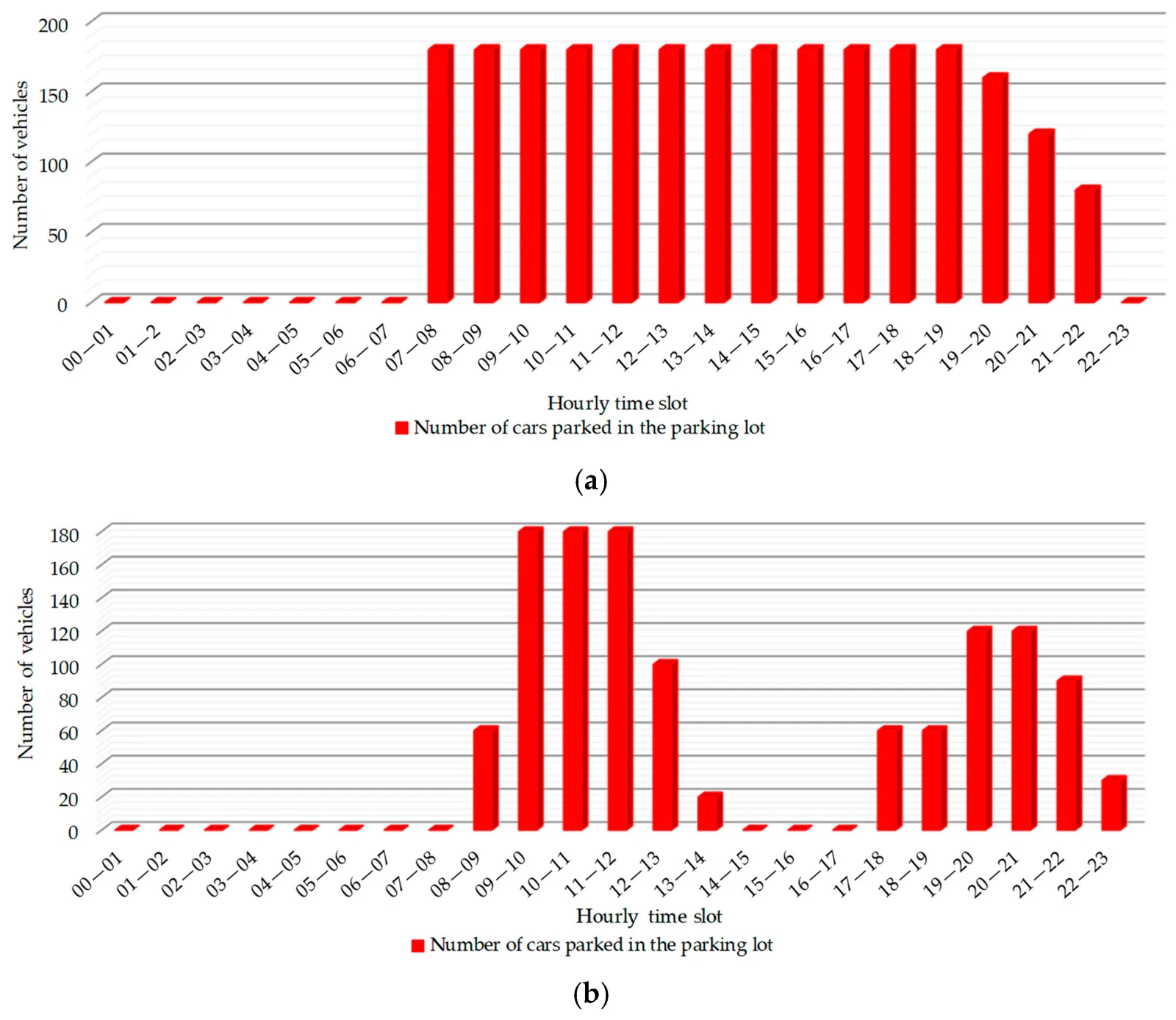

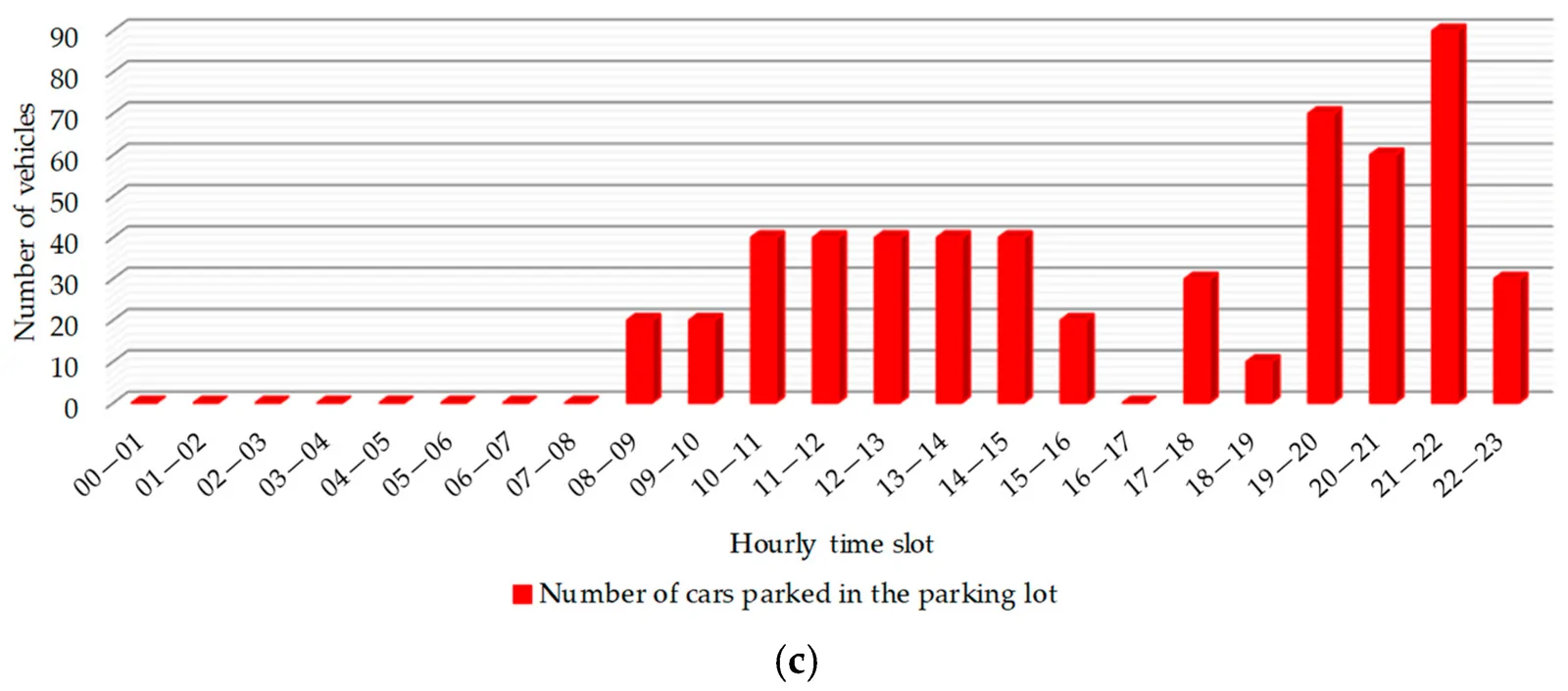
Estimated hourly car park occupancy rate. (**a**) School activity period; (**b**) sports/cultural event period; (**c**) holiday period.

Figure 6 presents the estimated hourly car park occupancy rate for typical periods (school activity, sports/cultural events, holidays).

Using the data in Figure 5 and Figure 6, the time intervals for motor vehicles that are operating or on stationary modes generating polluting emissions were identified. For these intervals, the total emissions on each level of the car park were calculated.

**Figure 7:**
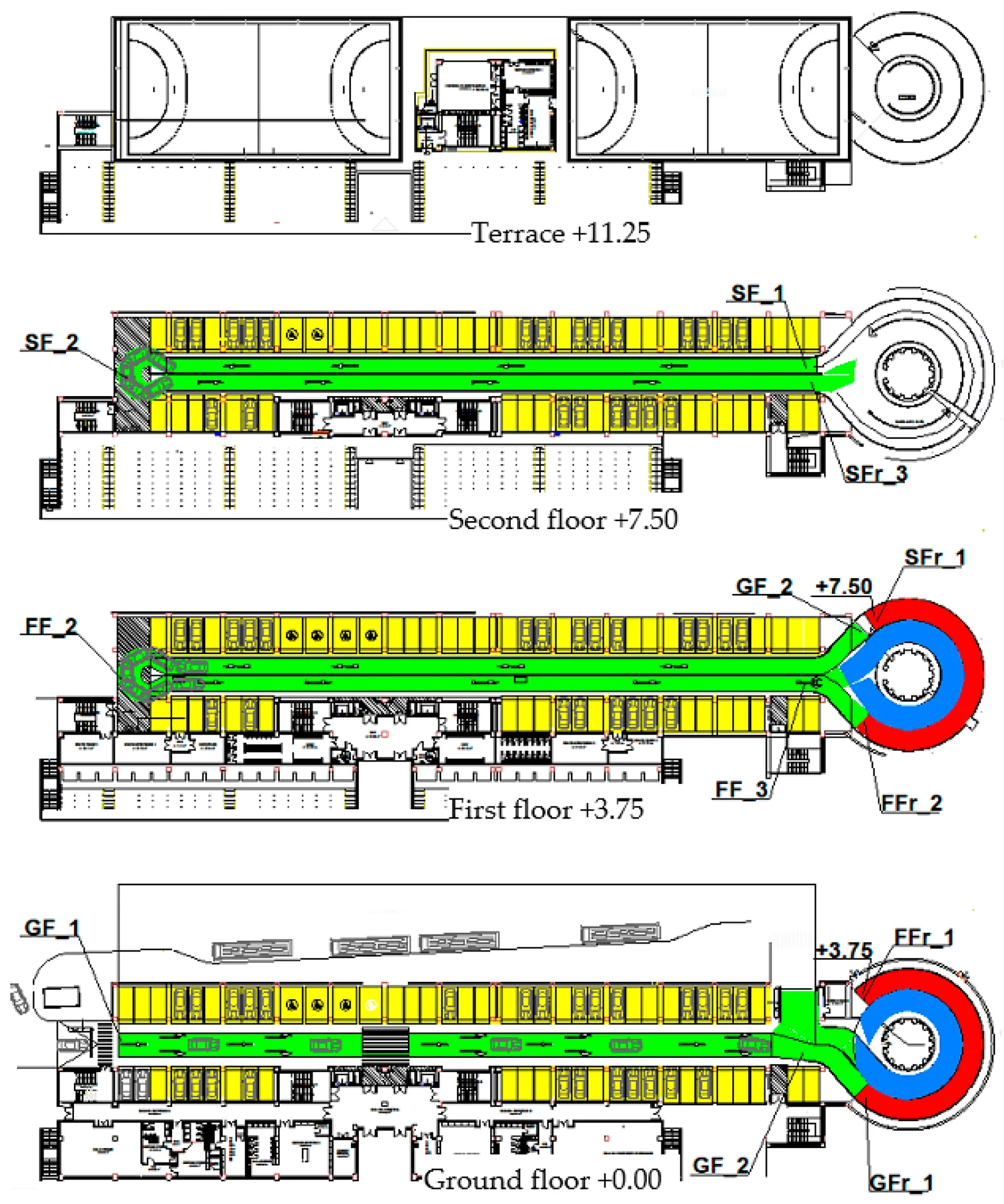
Parking emission zones. Yellow: Vehicle parking area. Green: Horizontal-vehicle-movement area. Red: Vehicle movement area on the ramp (ascending). Blue: Vehicle movement area on the ramp (descending).

In the first stage of analysis, areas with emissions specific to the mode of movement of vehicles (horizontal plan, uphill or downhill on a ramp) and parking spaces were identified and marked (Figure 7).

**Figure 8:**
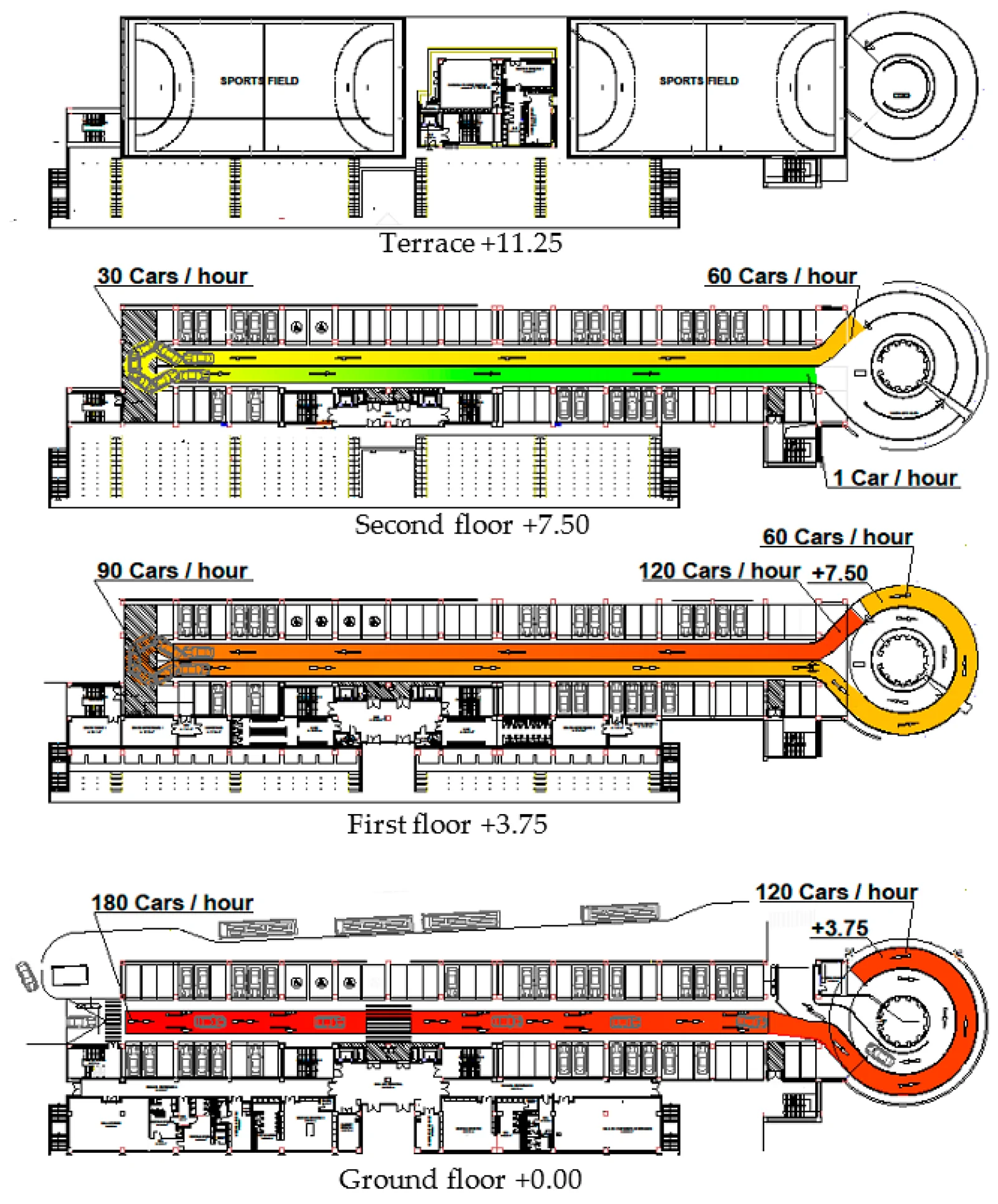
Density of the driving vehicles during the parking-lot-filling process; red—area with high traffic density; yellow—area with medium traffic density; green—area with low traffic density.

**Figure 9:**
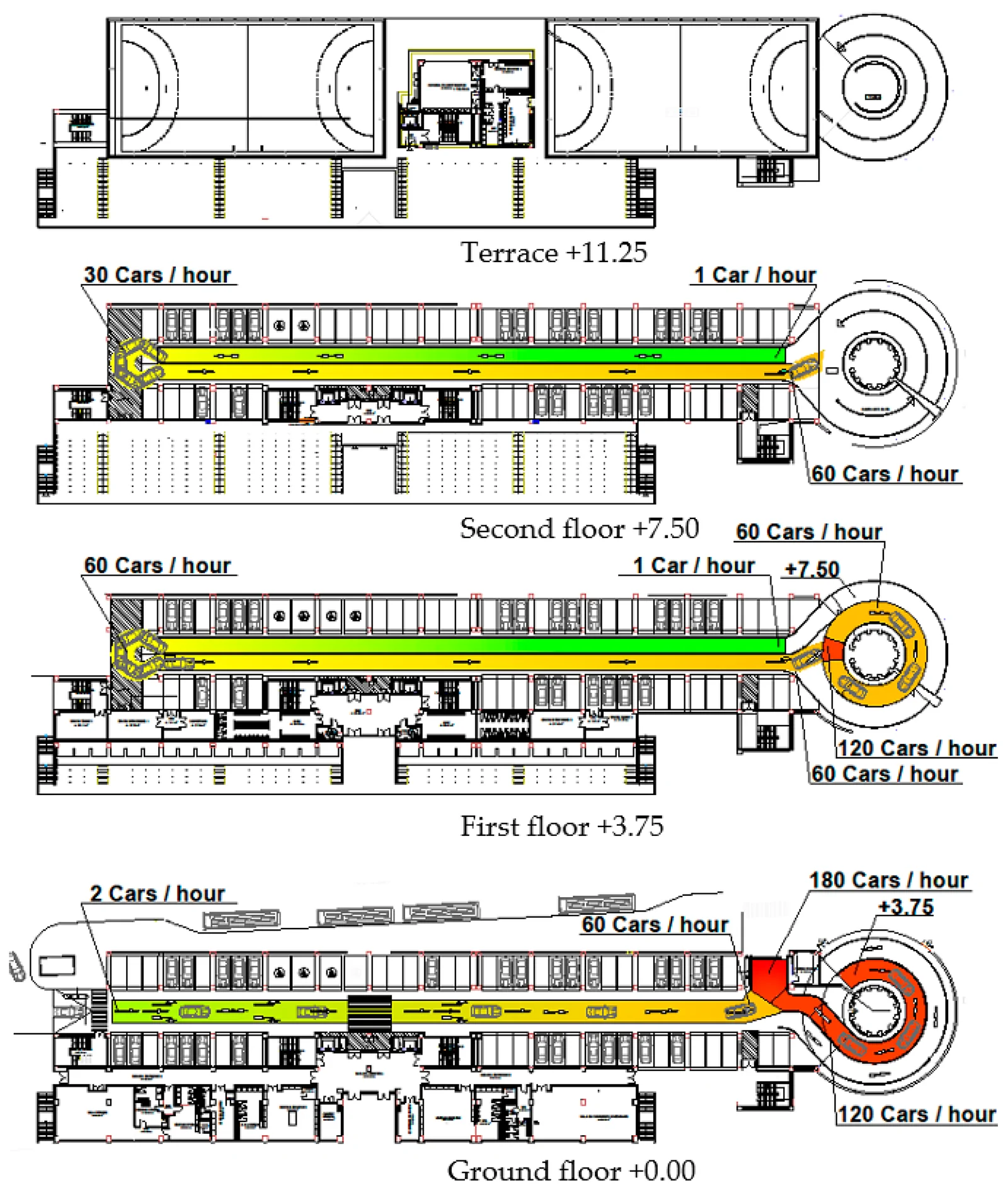
Density of the driving vehicles during the parking-lot-emptying process. Red—area with high traffic density; yellow—area with medium traffic density; green—area with low traffic density.

The maximum hourly traffic density considered for each parking level during the loading phase is represented in Figure 8, and during the emptying period it is represented in Figure 9. The color transition indicates the variation in the density of the number of motor vehicles transiting each level of the building.

**Figure 10:**
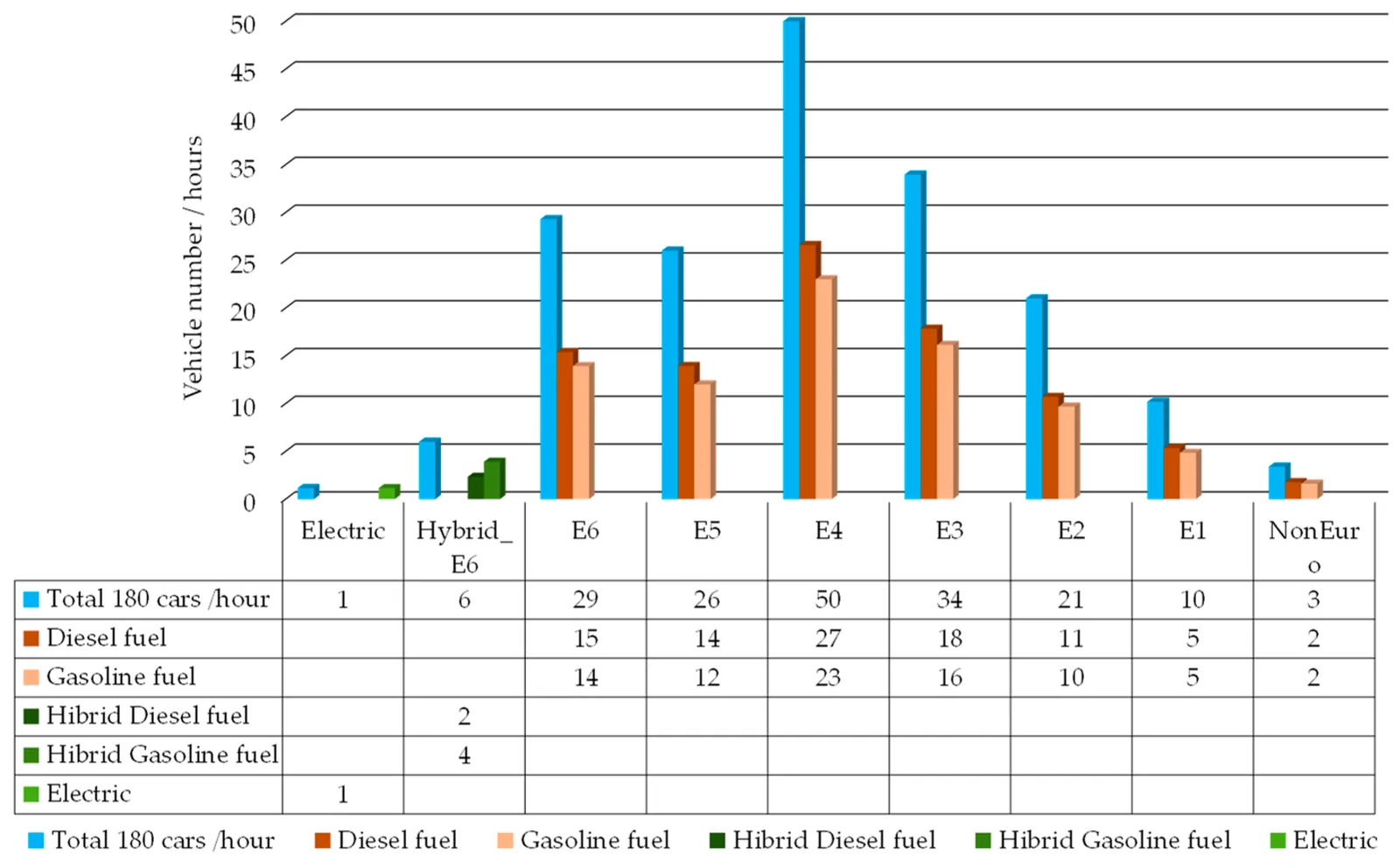
Hourly distribution by engine types and pollution standards for S1.

The first scenario (S1) analyzed is the one related to the school activity period. In this scenario, between 7:00 a.m. and 8:00 a.m., there is an intense activity of occupying parking spaces in which all 180 vehicles move inside the car park with hot engines to find a parking space. The average distance driven by a motor vehicle on a horizontal plane is 98.87 m on the ground floor, and for the total number of 180 motor vehicles the total distance is 17.79 km. Of the 180 vehicles, it is considered that 60 will park on the ground floor, and the remaining 120 will continue their journey to the first level to find a parking space. For the scenario S1, the hourly distribution of vehicles entering the car park is represented in Figure 10 by engine types and pollution standards. On the first floor the average distance driven by a motor vehicle on a horizontal plane is 153.28 m, and for the total number of 120 motor vehicles the total distance is 18.39 km. For the second floor the average distance driven by a motor vehicle on a horizontal plane is 106.57 m, and for the total number of 60 motor vehicles the total distance is 6.39 km. The distance driven by a motor vehicle up a ramp with a 10% slope is 62 m between the ground floor and the first floor, and the total distance is 7.44 km. The distance driven by a motor vehicle up a ramp with a 10% slope is 62 m between first floor and second floor, and the total distance is 3.72 km. In this scenario, given that vehicles enter the car park with a hot engine and there are no cold engine starts, the outside temperature does not influence emissions. In practice, the scenario is valid in the outside temperature range between −10 °C and 25 °C with the exception of NMVOC where we also have fugitive emissions from the evaporation process of gasoline from the tanks of parked vehicles for which the maximum scenario temperature was considered at 25 °C.

**Figure 11:**
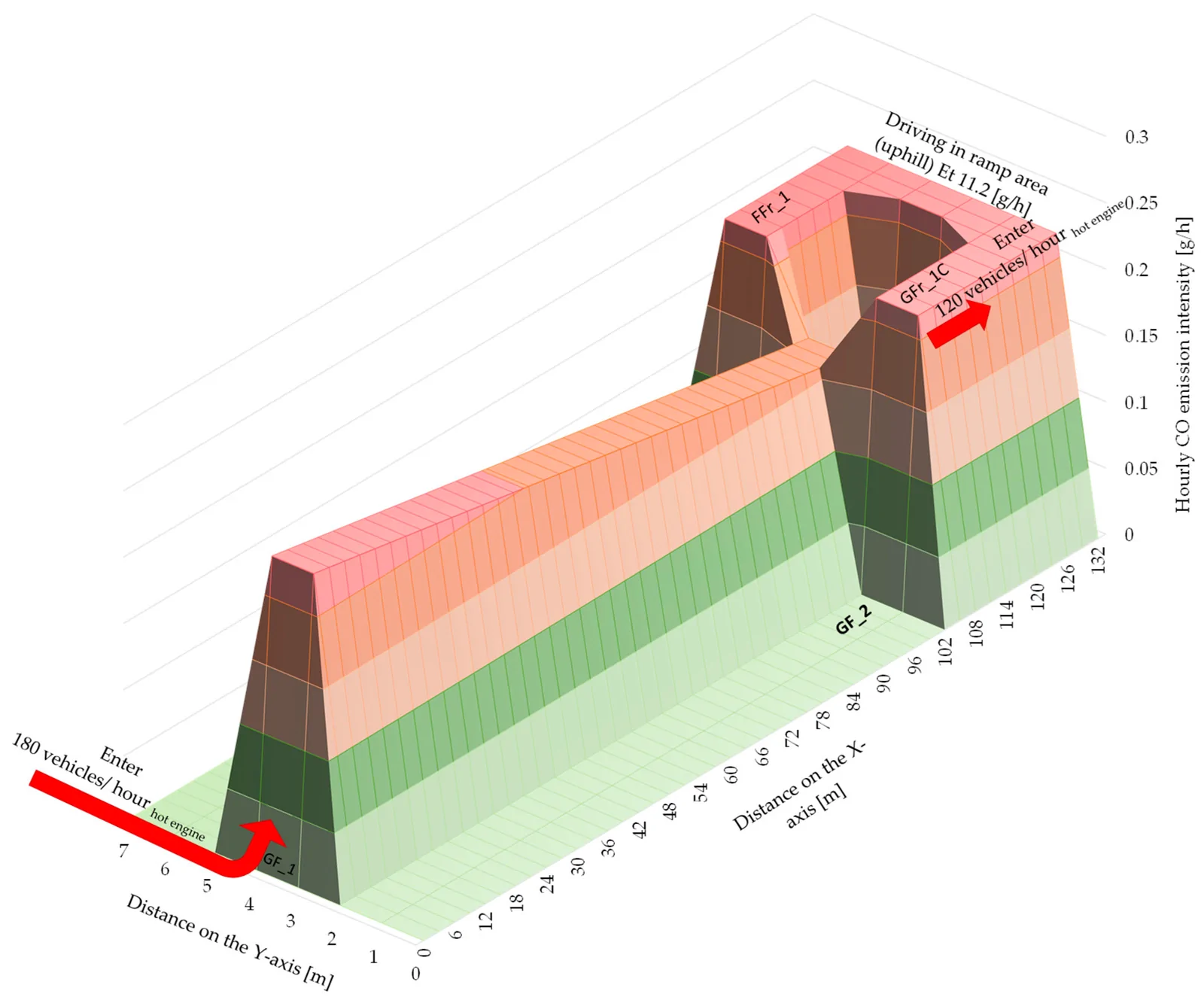
The CO emissions from the ground floor—S1.

**Figure 12:**
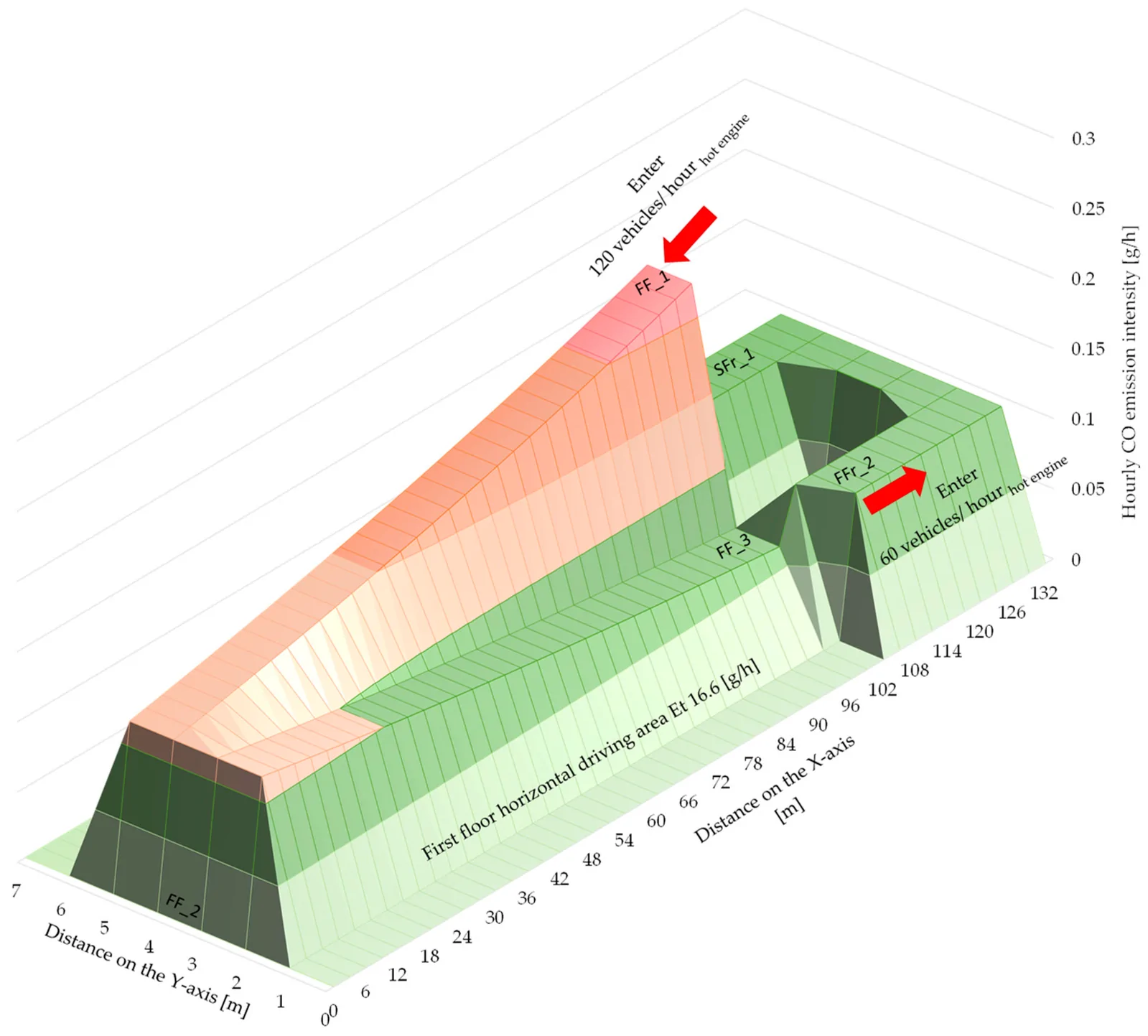
The CO emissions from the first floor—S1.

**Figure 13:**
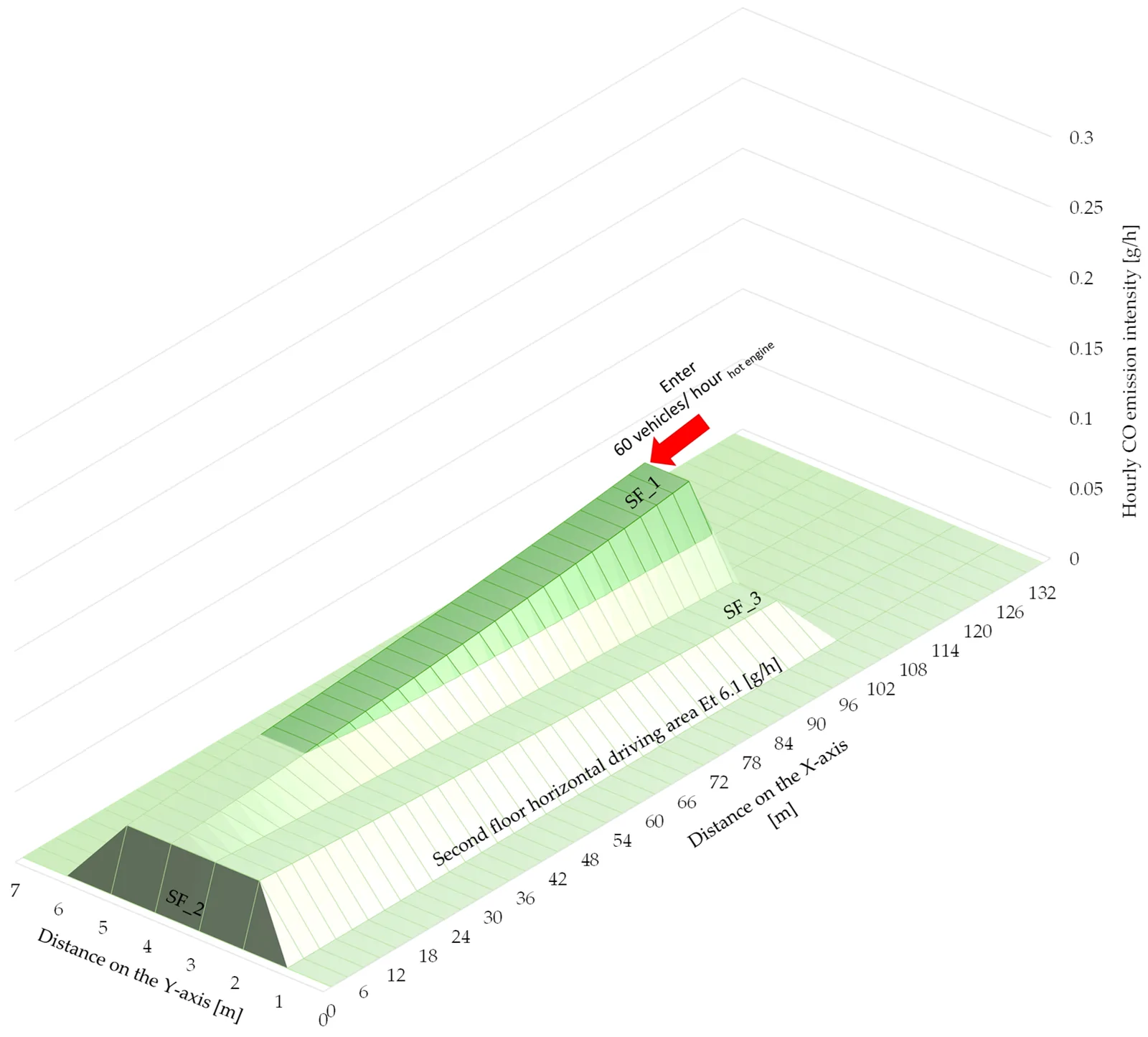
The CO emissions from the second floor—S1.

The CO emissions are presented graphically for each level in Figure 11, Figure 12 and Figure 13 in order to highlight the spatially distributed hourly emission gradient. On the graph, for each level, average hourly emission values were noted.

The calculated values for the total hourly emissions of all analyzed species (CO, CO_2_, NMVOC, NOx, NH_3_) are presented in Table 8 for each area.

Thus, Table 8 presents the results obtained from the calculations for Scenario 1, in which the representative areas in terms of maximum hourly emissions for the analyzed noxious species can be observed. From this point of view, it can be noted that for all analyzed noxious categories CO, CO_2_, NMVOC, NOx and NH_3_, the maximum calculated values are related to the first level of the MB-UPT car park. The results obtained are due to the fact that in this area vehicles travel an average distance greater than on the other levels to occupy a parking space, respectively to access the second level of the car park.

Figure 11, Figure 12 and Figure 13 present the spatial distributions of the average emissions in the analyzed areas. Regarding the local average emissions, in the flat area on the ground floor a decreasing variation with a linear evolution (from 0.23 g/h to 0.15 g/h) is observed from the GF_1 to GF_2 area (ramp entrance) due to the decrease in the number of traveling vehicles (from 180 to 120). Analyzing the following characteristic area represented by the ramp from the ground floor to the first floor, from GFr_1C to FFr_1, it is observed that in this area the average emissions have relatively constant values (of about 0.22 g/h) due to the fact that the ramp area is traversed by the 120 vehicles. Analyzing strictly the CO emissions presented graphically (Figure 11) it is observed that the average values in the ramp area are of the same order of magnitude as those at the parking entrance. When vehicles travel on the first level (the analysis is shown in Figure 12), a linear decreasing distribution of CO emissions is observed on the route FF_1–FF_2–FF_3 due to the decrease in the number of vehicles traveling (from 120 to 60), with values that vary between 0.22 g/h and 0.06 g/h. In the ramp area from FFr_1 to SFr_1, it is observed that the average emissions have relatively constant values (of about 0.10 g/h) due to the fact that the ramp area is traveled by the 60 vehicles. In the flat area on the second floor, a decreasing variation with a linear evolution (from 0.08 g/h to 0.02 g/h) is observed on the route SF_1–SF_2–SF_3 due to the decrease in the number of vehicles traveling (from 60 to one).

Analyzing the values in Table 8, it is observed that the CO_2_, NOx and NH_3_ emission species also have a similar evolution to CO, with the exception of the NMVOC species which has a different trend. The explanation is due to the fact that NMVOC emissions are not predominantly caused by the traveling of vehicles or the ramp multiplication coefficient, but are generally caused by evaporative emissions from the tanks of parked vehicles.

**Figure 14:**
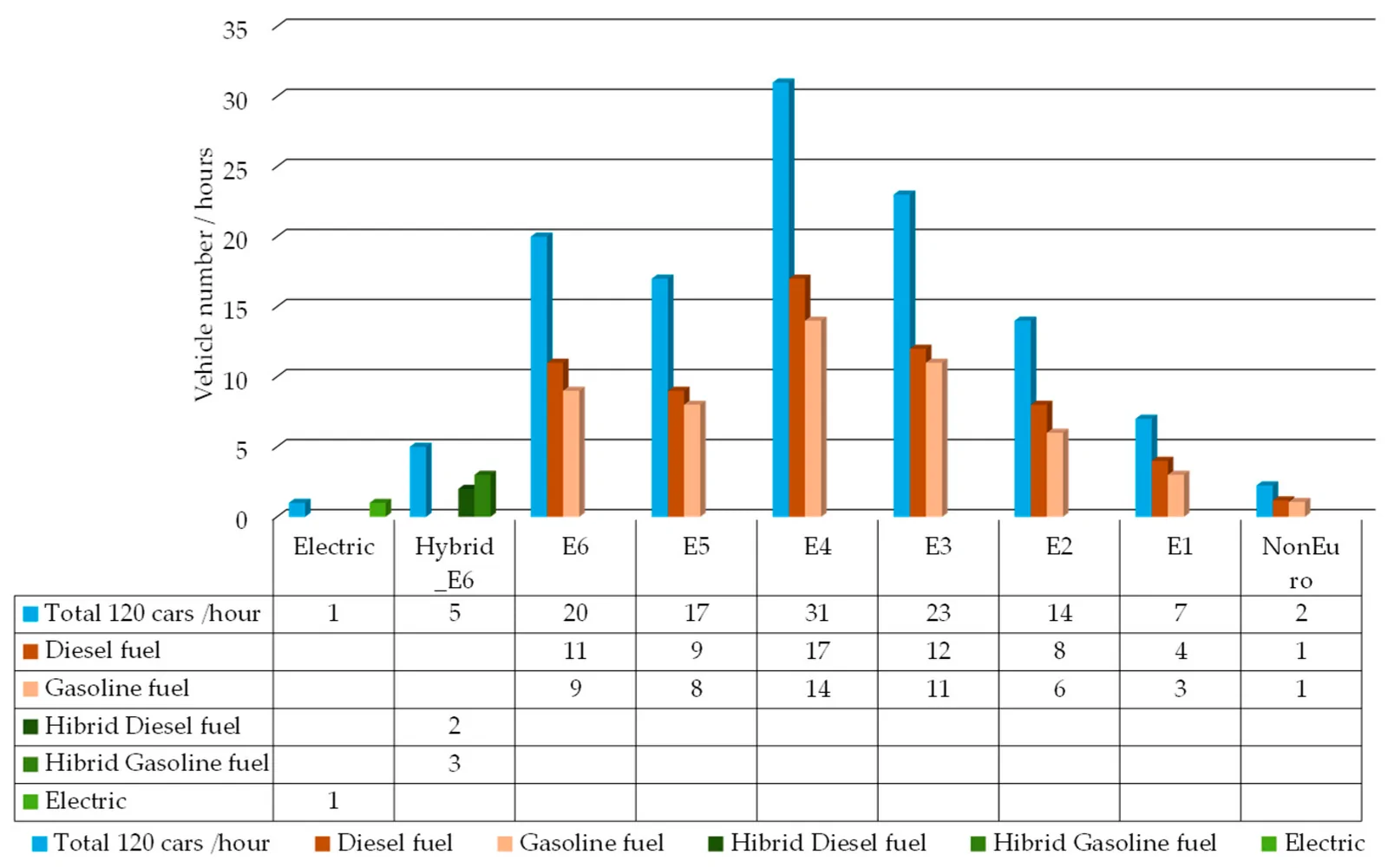
Hourly distribution by engine types and pollution standards for S2.

The second maximal scenario (S2) analyzed represents the scenario related to the school activity period in the cold season in which in the time interval 15.00 p.m.−16.00 p.m. there is an intense activity of leaving the parking spaces (approximately 120 vehicles start their engines and move inside the car park, with a cold engine). Inside the car park, between 3:00 PM and 4:00 PM, the estimated minimum temperature is 0 °C (the most common situation during the cold season in the city of Timisoara) and is the temperature considered in this scenario for emission factor calculation. For the scenario S2, the hourly distribution of vehicles leaving/entering the parking lot is represented in Figure 14 by engine types and pollution standards.

The average distance driven by a motor vehicle on a horizontal plane is 51.61 m on the ground floor, and for the total number of 40 motor vehicles the total distance is 2.06 km. On the first floor the average distance driven by a motor vehicle on a horizontal plane is 101.56 m, and for the total number of 40 motor vehicles the total distance is 4.06 km. For the second floor the average distance driven by a motor vehicle on a horizontal plane is 101.56 km, and for the total number of 40 motor vehicles the total distance is 4.06 km. The average distance driven by a motor vehicle downhill on a ramp with a 10% slope is 62 m between the second floor and the first floor, and the total distance is 2.48 km. The average distance driven by a motor vehicle downhill on a ramp with a 10% slope is 62 m between the first floor and the ground floor, and the total distance is 4.96 km. Also in this scenario, between 15.00 p.m. and 16.00 p.m., there is also an activity of occupying the vacated parking spaces in which another 120 vehicles move inside the parking lot with a hot engine to find a parking space. The average distance driven by a motor vehicle on a horizontal plane is 64.42 m on the ground floor, and for the additional number of 120 vehicles the total distance is 7.73 km. On the first floor, the average distance driven by a motor vehicle on a horizontal plane is 153.28 m, and for the additional number of 80 motor vehicles the total distance is 12.6 km. For the second floor, the average distance driven by a motor vehicle on a horizontal plane is 106.57 m, and for the additional number of 40 motor vehicles the total distance is 4.26 km.

The average distance driven by a car uphill on a ramp with a 10% slope is 62 m between the ground floor and the first floor, and the total distance is 4.96 km. The average distance driven by a motor vehicle uphill on a ramp with a 10% slope is 62 m between the first floor and the second floor, and the total distance is 2.48 km.

Given that in scenario S2 the effects of two flows of use of the BMB-UPT car park overlap (vehicles leaving/occupying the car park), noxious emissions accumulate in the car park from vehicles entering the parking lot with a hot engine and from starting and driving with a cold engine (0 °C) for those leaving the car park.

**Figure 15:**
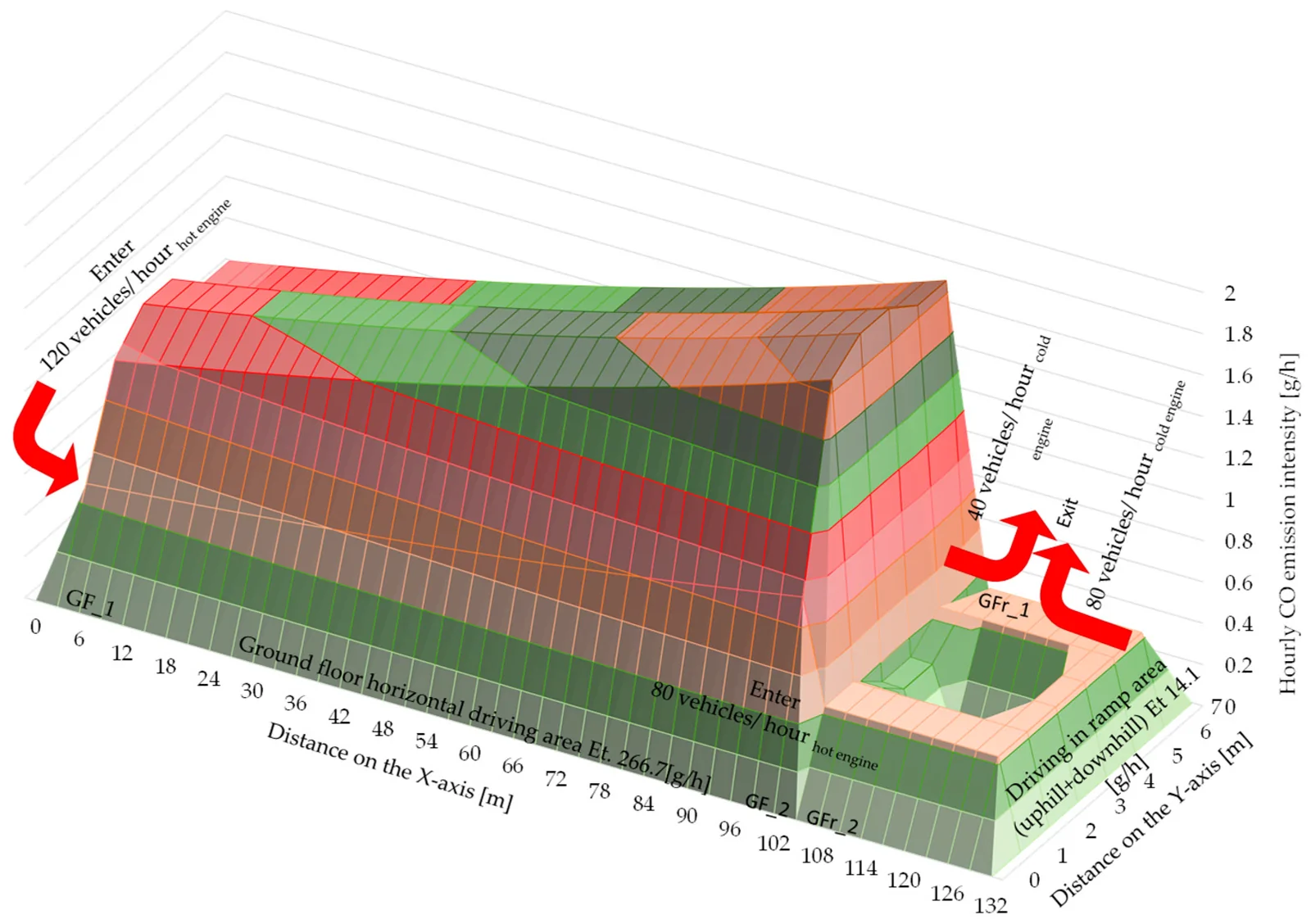
The CO emissions from the ground floor—S2.

**Figure 16:**
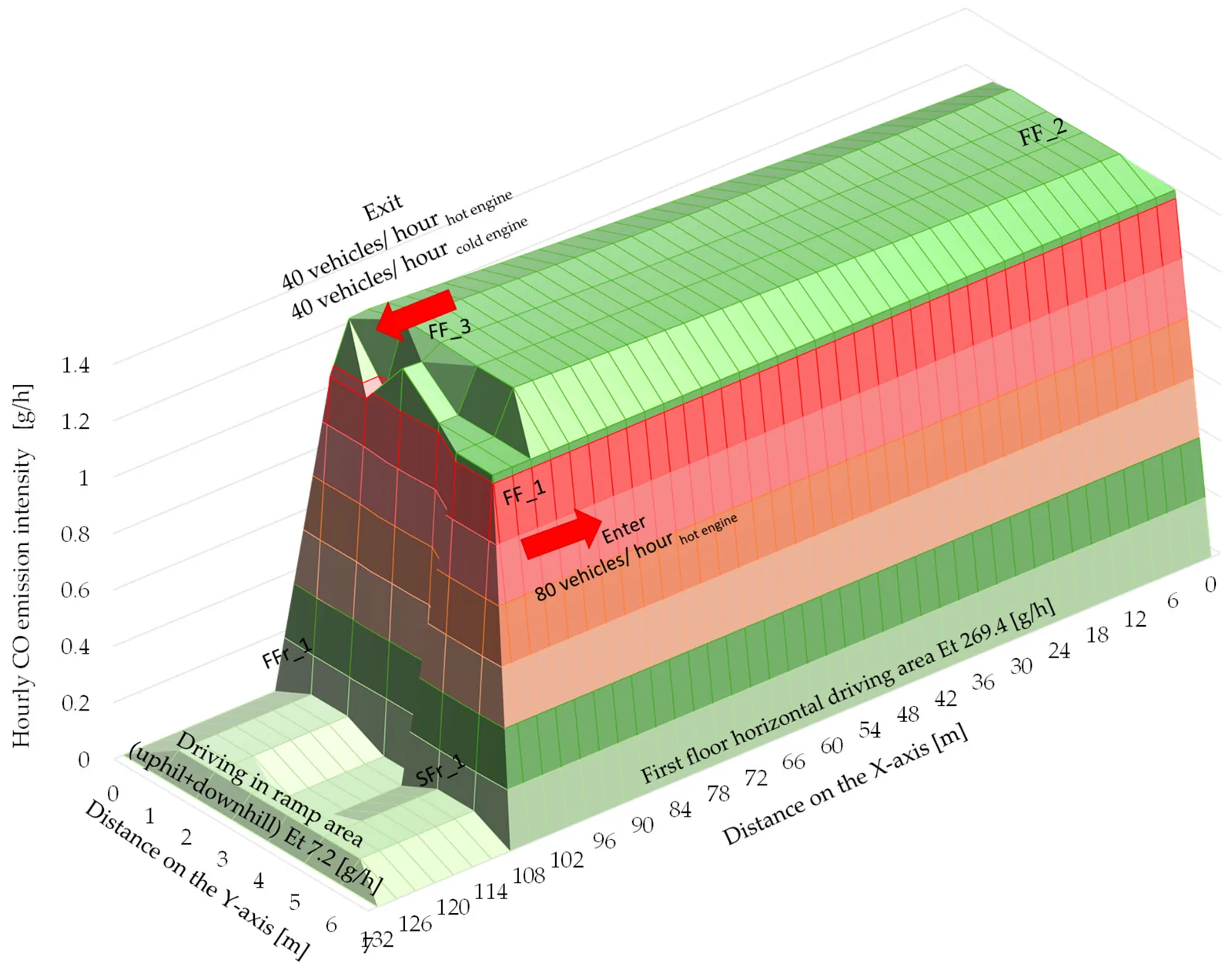
The CO emissions from the first floor—S2.

**Figure 17:**
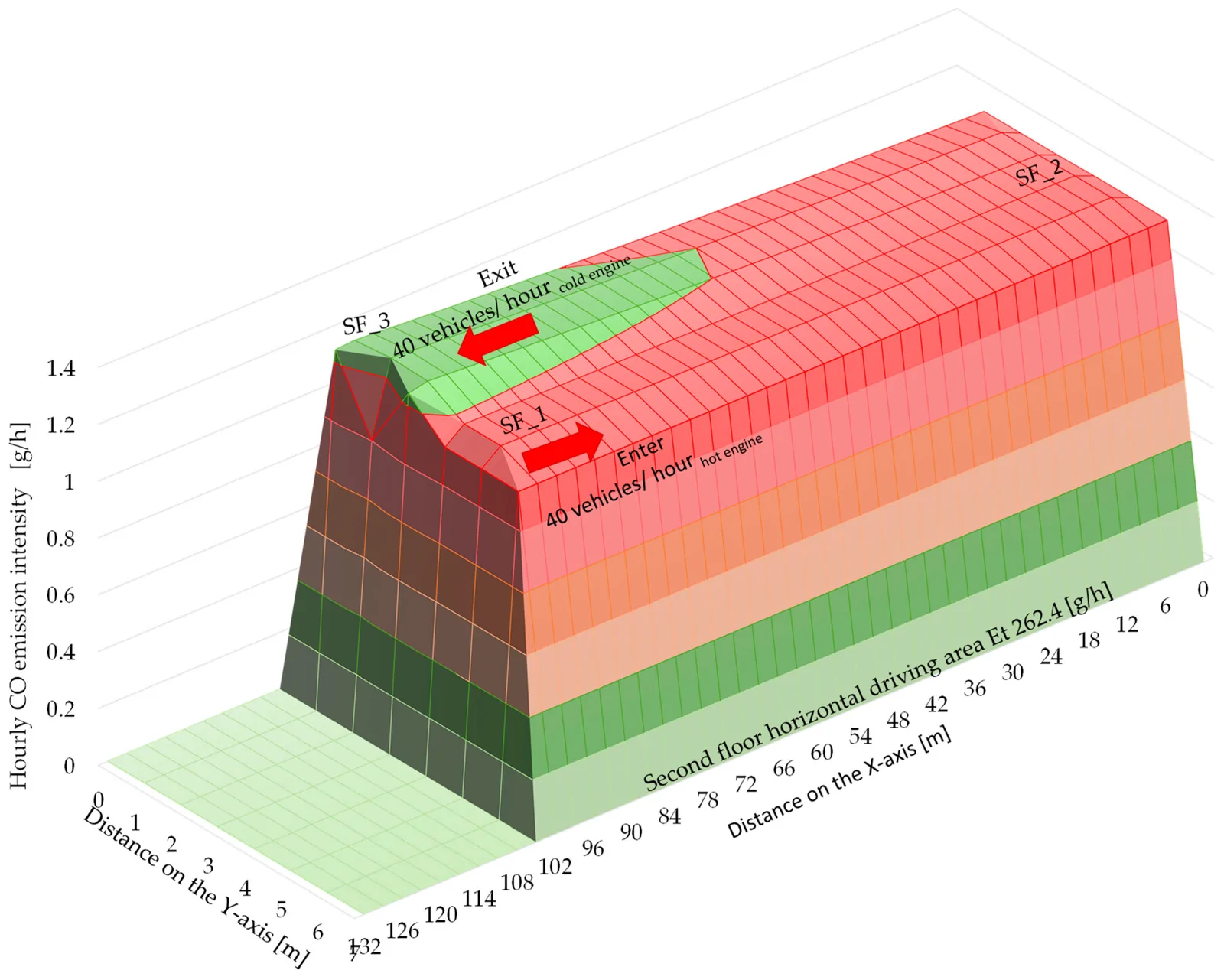
The CO emissions from the second floor—S2.

The CO emissions are presented graphically for each level in Figure 15, Figure 16 and Figure 17 in order to highlight the spatially distributed hourly emission gradient. On the graph, for each level, average hourly emission values were noted.

The calculated values for total hourly emissions of all analyzed species (CO, CO_2_, NMVOC, NOx, NH_3_) are presented in Table 9 for each area.

Table 9 presents the results obtained from the calculations for Scenario 2, in which the representative zones in terms of maximum hourly emissions for the analyzed noxious species can be observed. From this point of view, it can be noted that for all analyzed noxious categories CO, CO_2_, NMVOC, NOx and NH_3_, the maximum calculated values are related to first level of the MB-UPT car park. The results obtained are due to the fact that in this zone, vehicles travel an average distance greater than on the other levels to occupy a parking space, respectively to access the second level of the parking. Additionally, a parking-lot-unloading process takes place, in which 40 vehicles start and move in order to leave the parking spaces with cold engines.

In terms of local average emissions, in the flat area on the ground floor, an increasing variation is observed (from 0.95 g/h to 1.96 g/h) from area GF_1 to GF_2 (ramp entrance) due to the overlapping effects of the hourly flows of use of the BMB-UPT parking lot at a temperature of 0 °C (40 vehicles leaving the car park with cold engines and respectively entrance and traveling with hot engines—120 vehicles). The predominant factor of the emissions generated is the cold engine start. Analyzing the next characteristic area represented by the ramp from the ground floor to the first floor, from GFr_1C to FFr_1, it is observed that in this area the average emissions have relative constant values of about 0.43 g/h, due to the cumulative effect of the 80 vehicles that leave the car park down the ramp with a cold engine and the 80 vehicles that climb the ramp with a hot engine to park. When the vehicles travel on the first level (the analysis is shown in Figure 16), a distribution of CO emissions with values between 1.38 g/h and 1.23 g/h is observed on the route FF_1–FF_2–FF_3 due to the traveling vehicles (80 enter and 80 leave: 40 with a cold engine and 40 with a hot engine). At points FF_1 and FF_3, maximum values are recorded due to the ramp effect. In the ramp area from FFr_1 to SFr_1, it is observed that the average emissions have relatively constant values (of about 0.06 g/h) due to the fact that the ramp area is traveled by the 40 vehicles climbing the ramp and 40 vehicles driving down the ramp. In the flat area on the second floor, an increasing variation with a linear evolution (from 1.12 g/h to 1.26 g/h) is observed on the route SF_1–SF_2–SF_3 due to the 40 vehicles traveling with cold engines to leave the parking lot and the 40 vehicles traveling with hot engines to occupy a parking space. At points SF_1 and SF_3, maximum values are recorded due to the ramp effect.

Analyzing the values in Table 9, it is observed that the CO_2_, NOx and NH_3_ emission species also have a similar evolution to CO, with the exception of the NMVOC species which has a different trend. The explanation is due to the fact that NMVOC emissions are predominantly caused by cold engine starts.

**Figure 18:**
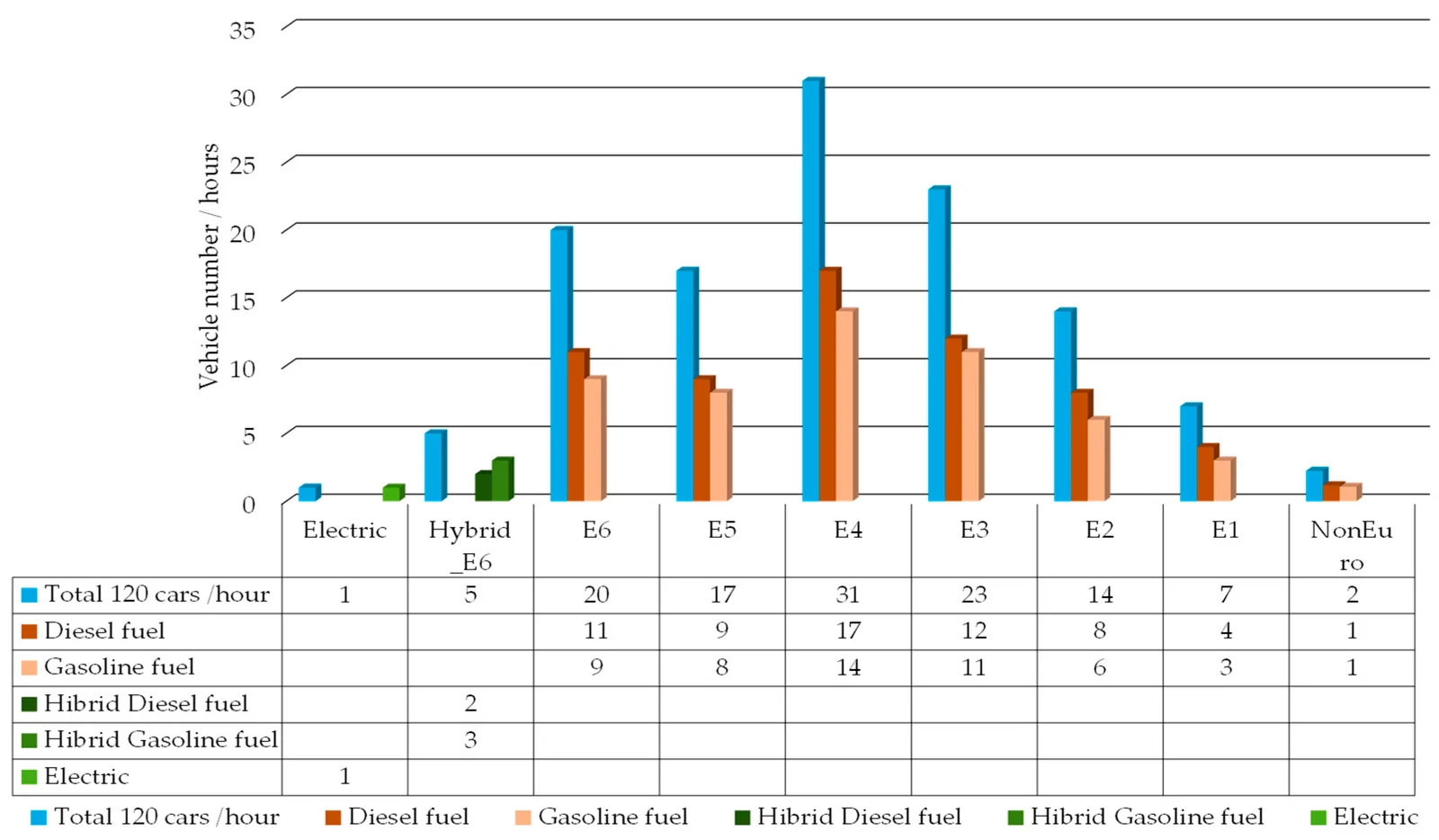
Hourly distribution by engine types and pollution standards for S3.

The third scenario (S3) analyzed is the one related to the school activity period in the summer season (temperatures above 25 °C with an maximum of 35 °C) in which a maximum of the phenomenon of evaporation of NMVOC from the tanks of parked motor vehicles is recorded. These emissions overlap with the emissions generated by the normal activity of the car park. In this scenario, in the time interval 15.00 p.m.−16.00 p.m. there is an intense activity of emptying the parking lot (120 vehicles start their engines and move inside the parking lot, with a cold engine at a temperature of 35 °C). For the scenario S3, the hourly distribution of vehicles leaving/entering the parking lot is represented in Figure 18 by engine types and pollution standards. The average distance driven by a motor vehicle on a horizontal plane is 51.61 m on the ground floor, and for 40 motor vehicles the total distance is 2.06 km. On the first floor the average distance driven by a motor vehicle on a horizontal plane is 101.56 m, and for 40 motor vehicles the total distance is 4.06 km. For the second floor the average distance driven by a motor vehicle on a horizontal plane is 101.56 km, and for 40 motor vehicles the total distance is 4.06 km. The average distance driven by a motor vehicle downhill on a ramp with a 10% slope is 62 m between the second floor and the first floor, and the total distance is 2.48 km. The average distance driven by a motor vehicle downhill on a ramp with a 10% slope is 62 m between the first floor and the ground floor, and the total distance is 4.96 km. Also in this scenario, between 15.00 p.m. and 16.00 p.m., there is also an activity of occupying the vacated parking spaces in which another 120 vehicles move inside the parking lot with a hot engine to find a parking space. The average distance driven by a motor vehicle on a horizontal plane is 64.42 m on the ground floor, and for 120 vehicles the total distance is 7.73 km. On the first floor, the average distance driven by a motor vehicle on a horizontal plane is 153.28 m, and for 80 motor vehicles the total distance is 12.26 km. For the second floor, the average distance driven by a motor vehicle on a horizontal plane is 106.57 m, and for 40 motor vehicles the total distance is 4.26 km.

The average distance driven by a car uphill on a ramp with a 10% slope is 62 m between the ground floor and the first floor, and the total distance is 4.96 km. The average distance driven by a motor vehicle uphill on a ramp with a 10% slope is 62 m between the first floor and the second floor, and the total distance is 2.48 km.

**Figure 19:**
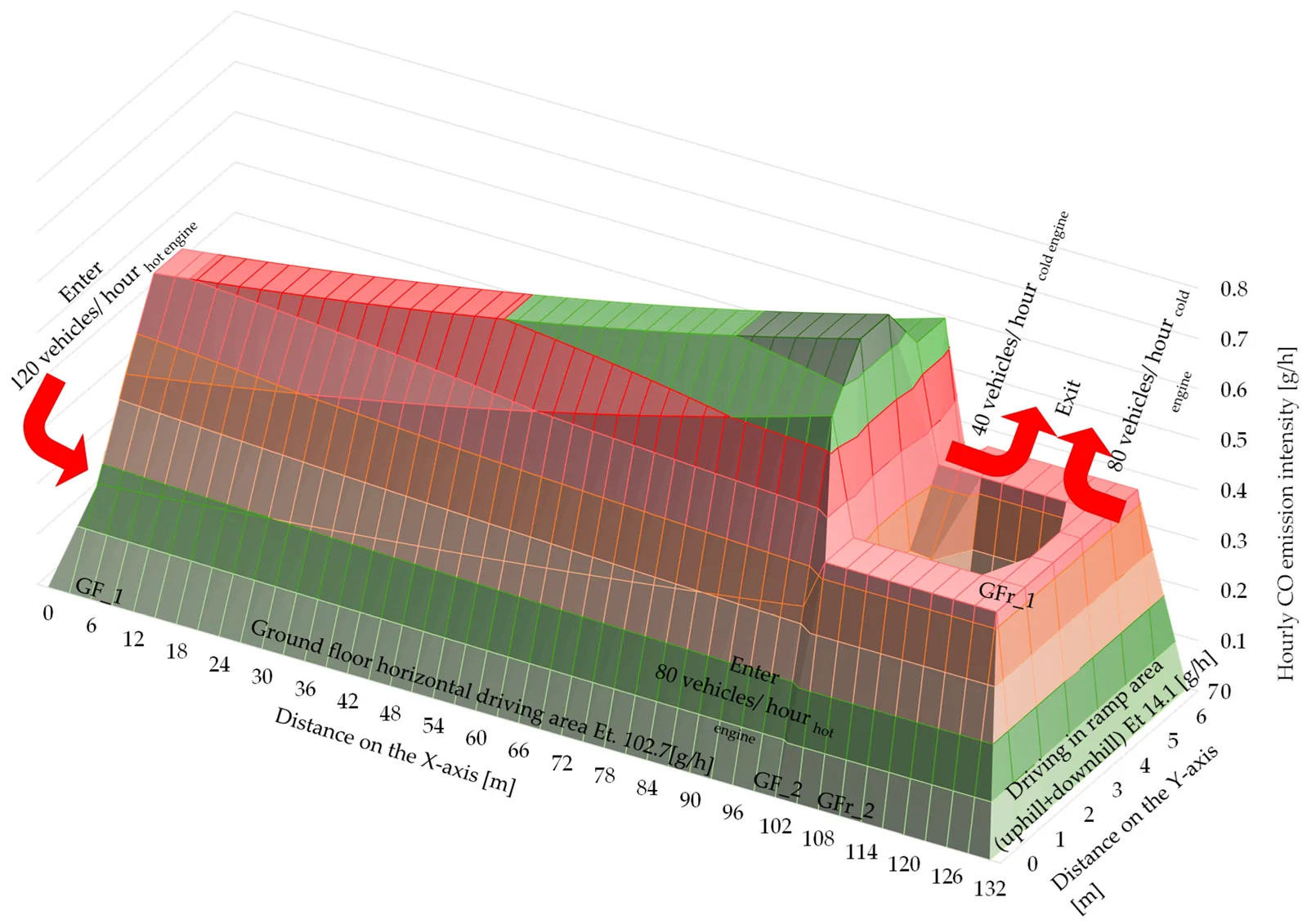
The CO emissions from the ground floor—S3.

**Figure 20:**
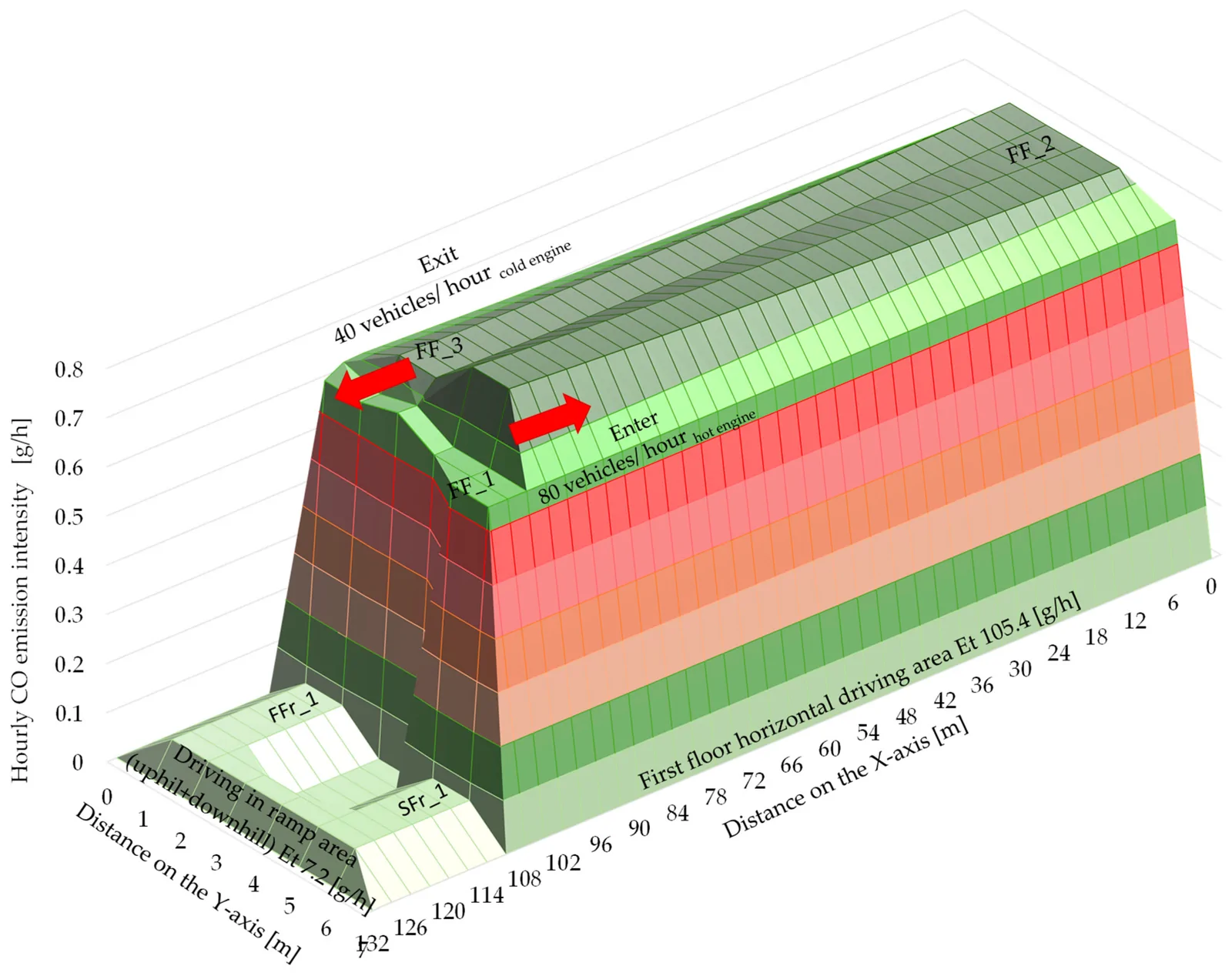
The CO emissions from the first floor—S3.

**Figure 21:**
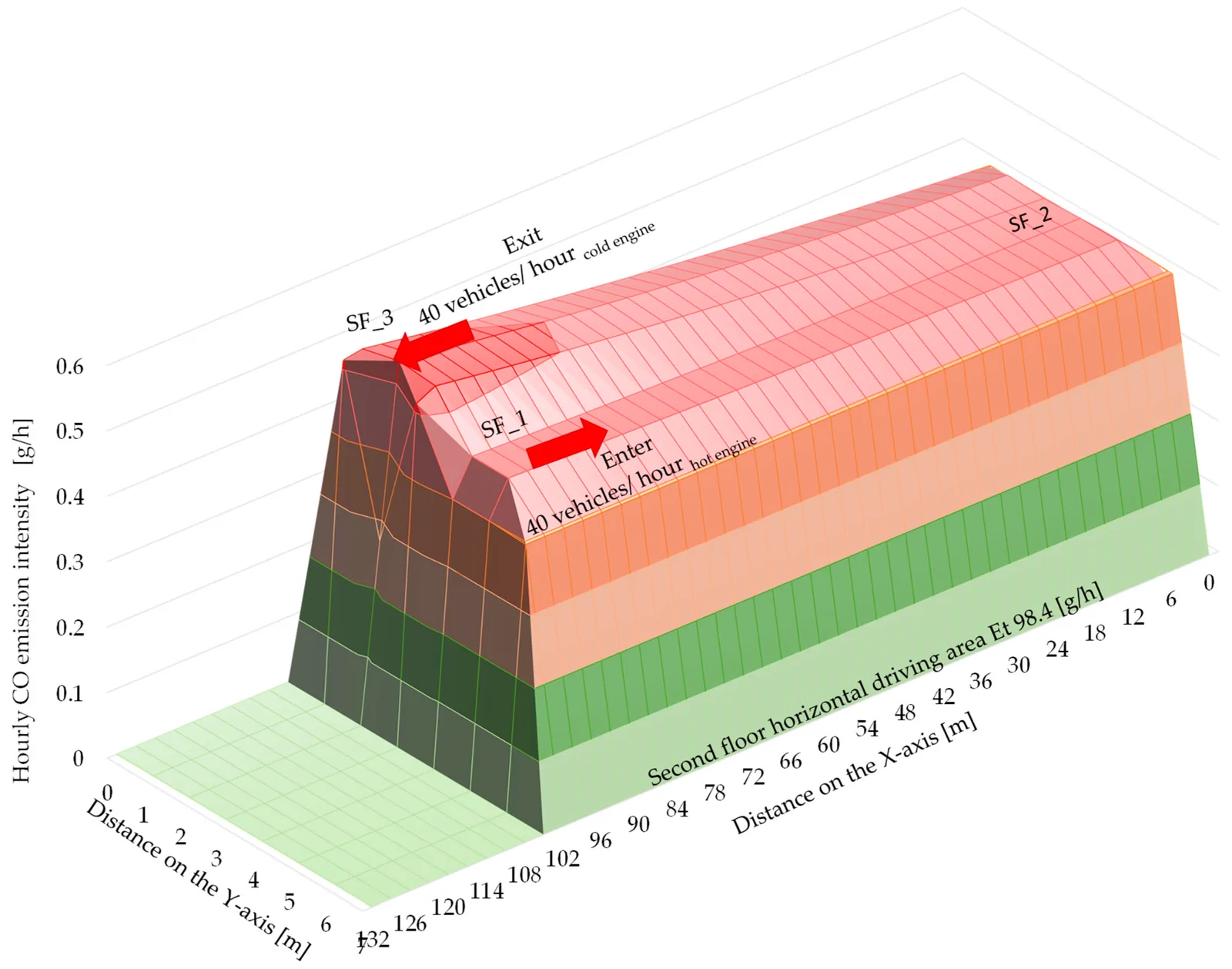
The CO emissions from the second floor—S3.

The CO emissions are presented graphically for each level in Figure 19, Figure 20 and Figure 21 in order to highlight the spatially distributed hourly emission gradient. On the graph, for each level, average hourly emission values were noted.

The calculated values for total hourly emissions of all analyzed species (CO, CO_2_, NMVOC, NOx, NH_3_) are presented in Table 10 for each area.

Thus, Table 10 presents the results obtained from the calculations for Scenario 3, in which the representative zones in terms of maximum hourly emissions for the analyzed noxious species can be observed. From this point of view, it can be noted that for all analyzed noxious categories CO, CO_2_, NMVOC, NOx, and NH_3_, the maximum calculated values are related to the first level of the MB-UPT car park. The results obtained are due to the fact that in this zone, vehicles travel a greater average distance than on the other levels to occupy a parking space on the first level and respectively for access to the second level of the car park.

Regarding the local average emissions, in the flat area on the ground floor, an increasing variation is observed (from 0.35 g/h to 0.76 g/h) from area GF_1 to GF_2 (ramp entrance) due to the overlapping effects of the hourly flows of use of the BMB-UPT car park at a temperature of 35 °C (40 vehicles leaving the parking lot with cold engines and respectively entering and circulating with hot engines—120 vehicles). The predominant factor of the emissions generated is the cold engine start. Analyzing the next characteristic area represented by the ramp from the ground floor to the first floor, from GFr_1C to FFr_1, it is observed that in this area the average emissions have relatively constant values of about 0.43 g/h, due to the cumulative effect of the 80 vehicles that leave the car park down the ramp with a cold engine and the 80 vehicles that climb the ramp with a warm engine to park. When vehicles travel on the first level (the analysis is shown in Figure 20), a distribution of CO emissions with values between 0.79 g/h and 0.64 g/h is observed on the route FF_1–FF_2–FF_3 due to the vehicles that are traveling (80 enter and 80 leave: 40 with a cold engine and 40 with a hot engine). At points FF_1 and FF_3, maximum values are recorded due to the ramp effect. In the ramp area from FFr_1 to SFr_1, it is observed that the average emissions have relatively constant values (of about 0.07 g/h) due to the fact that the ramp area is traveled by the 40 vehicles that climb the ramp and 40 vehicles that are driving down the ramp. In the flat area on the second floor, an increasing variation with a linear evolution (from 0.43 g/h to 0.45 g/h) is observed on the route SF_1–SF_2–SF_3 due to the 40 vehicles traveling with cold engines to leave the car park and the 40 vehicles traveling with hot engines to occupy a parking space. At points SF_1 and SF_3, maximum values are recorded due to the ramp effect.

Analyzing the values in Table 10, it is observed that the CO_2_, NOx and NH_3_ emission species also have a similar evolution to CO, with the exception of the NMVOC species which has a different trend. The explanation is due to the fact that NMVOC emissions are predominantly caused by cold engine starts combined with emissions from the tanks of parked vehicles.

The data presented in Table 8, Table 9 and Table 10 was centralized in Table 11 for a comparative analysis between the three scenarios S1–S3.

**Figure 22:**
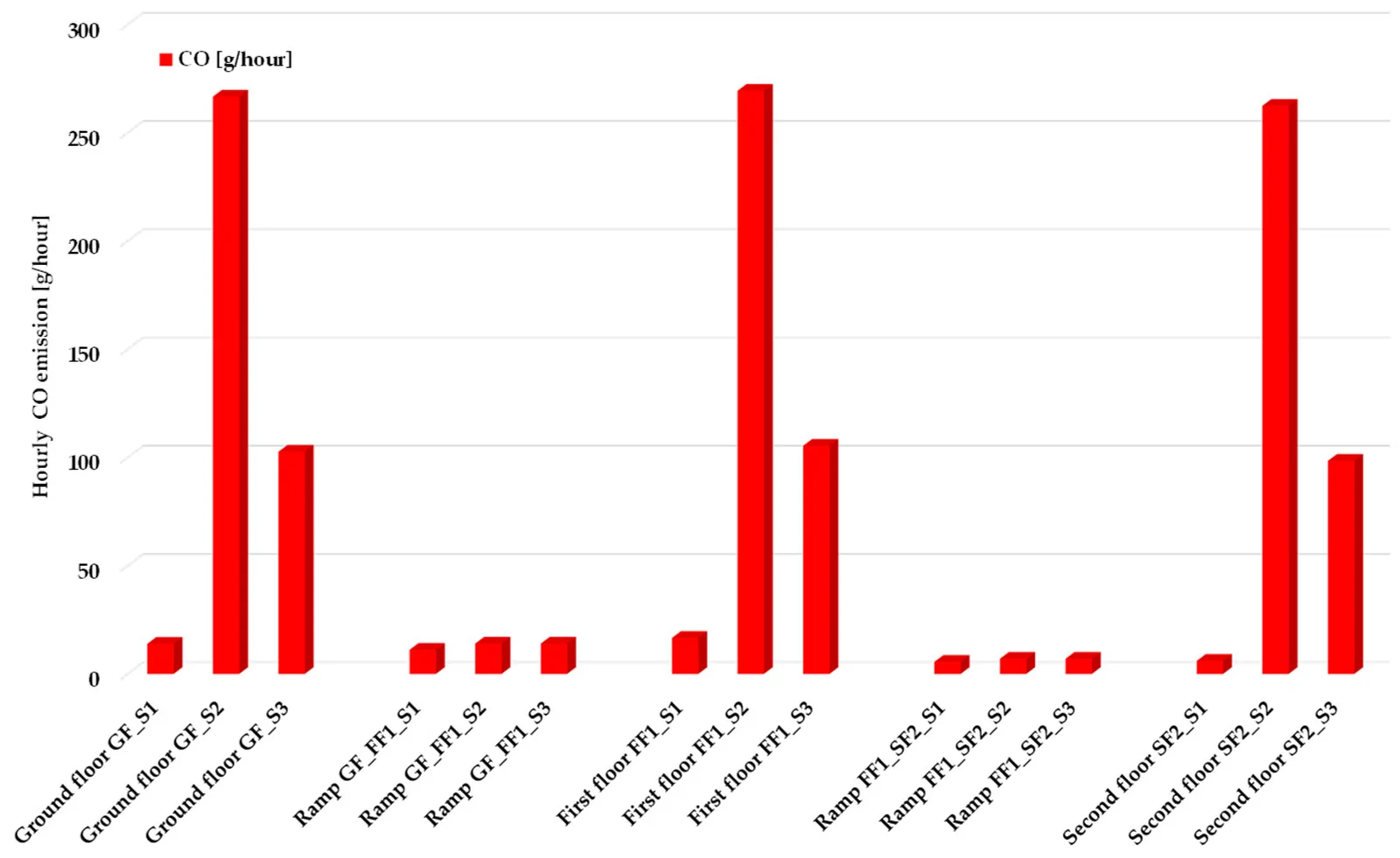
Comparative graph for total hourly emissions for CO, in the scenarios S1, S2 and S3 for all representative zones.

**Figure 23:**
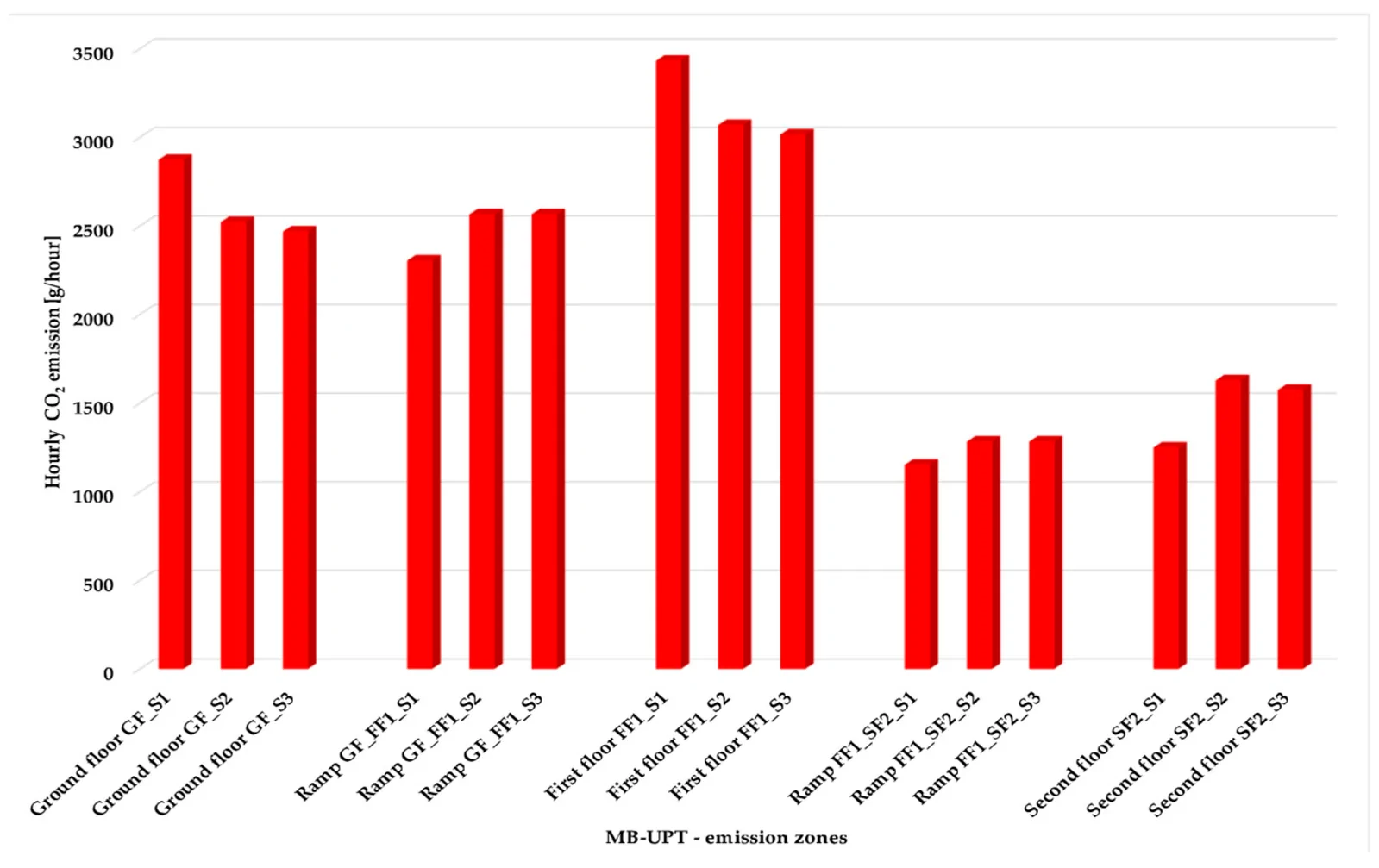
Comparative graph for total hourly emissions for CO_2_, in the scenarios S1, S2 and S3 for all representative zones.

**Figure 24:**
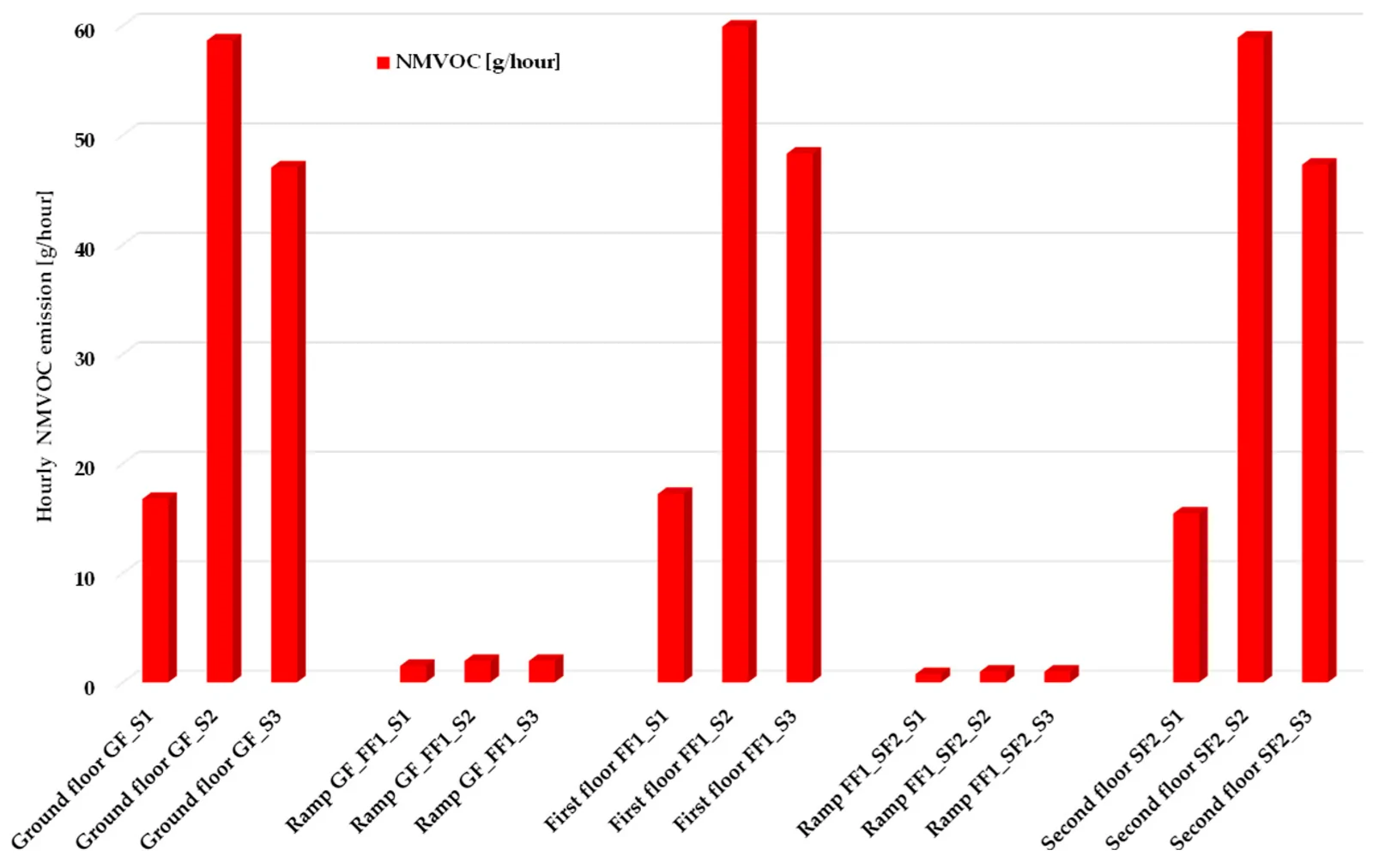
Comparative graph for total hourly emissions for NMVOC, in the scenarios S1, S2 and S3 for all representative zones.

**Figure 25:**
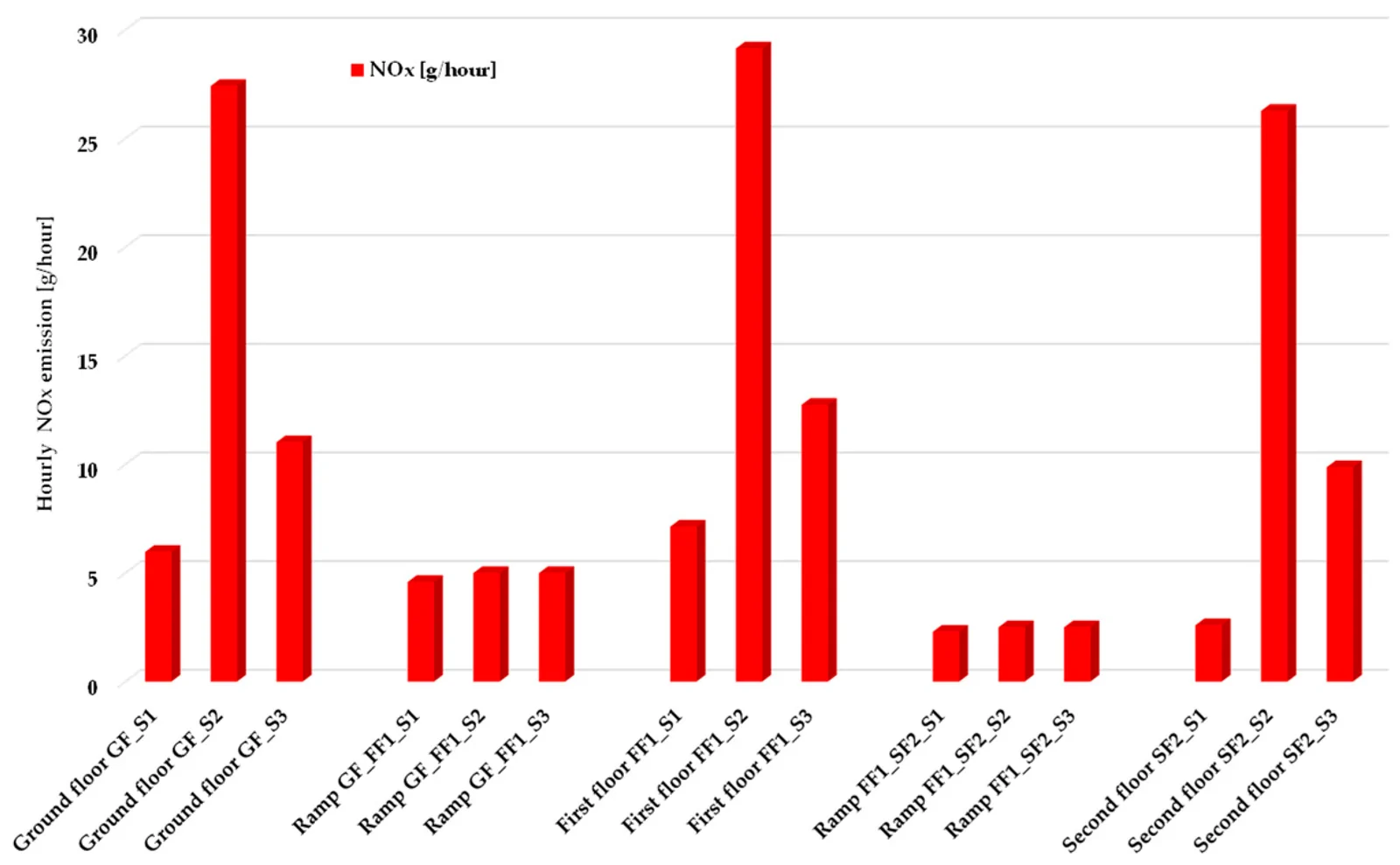
Comparative graph for total hourly emissions for NOx, in the scenarios S1, S2 and S3 for all representative zones.

**Figure 26:**
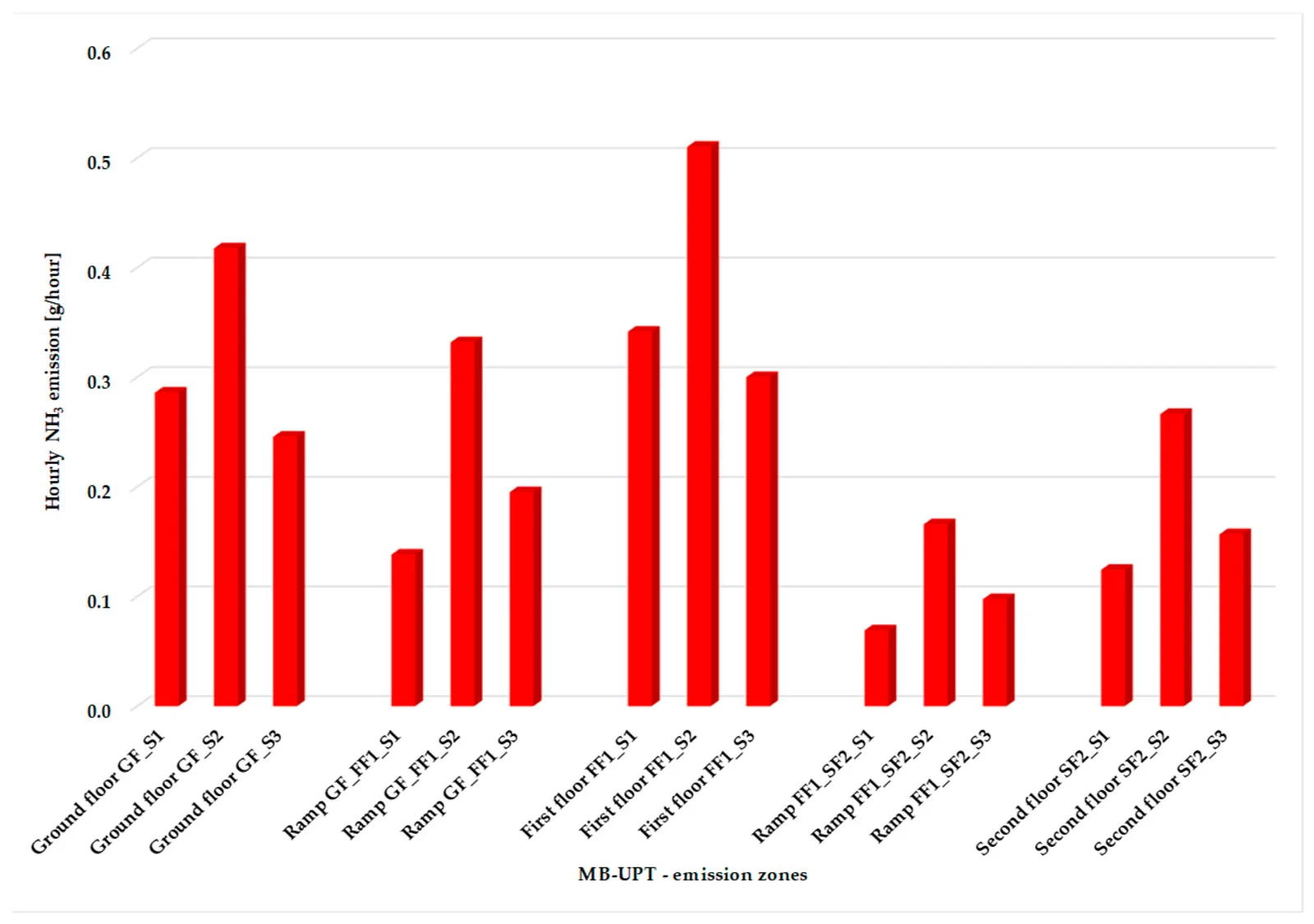
Comparative graph for total hourly emissions for NH_3_, in the scenarios S1, S2 and S3 for all representative zones.

The comparative analyses between the three scenarios for each species of gaseous pollutant are presented graphically in the following figures (Figure 22, Figure 23, Figure 24, Figure 25 and Figure 26).

Figure 22 graphically presents the results of the maximum hourly values of CO emissions for all representative zones related to the three scenarios (S1–S3).

In scenario S1, although vehicles travel a significant distance inside the car park and this fills to capacity (180 cars), emissions are minimal due to them travelling with engines that are already hot. The major difference occurs in scenarios S2 and S3, where the starting of vehicles leaving the car park becomes the critical factor. Thus, in the winter scenario S2 (0 °C) maximum hourly CO emissions (approx. 270 g/h) are generated due to cold engine start and operation of vehicles (rich fuel mixture). Similarly, the summer scenario S3 (35 °C) maintains the dynamics of S2, but CO emissions decrease by over 60% (to approx. 100 g/h) due to the higher ambient temperature which shortens the engine heat-up period. On the other hand, the almost-zero values recorded when vehicles travel on ramps in all three scenarios demonstrate that the easy travel between levels does not generate significant emissions because vehicles leaving the car park move on the ramp with engine braking, therefore with minimal emissions. Analysis of the graph highlights a direct correlation between the thermal regime at start-up of the engines and the volume of CO emissions.

The maximum hourly values of CO_2_ emissions for all representative zones related to the three scenarios (S1–S3) are graphically presented in Figure 23.

In all three scenarios, emissions are highest on the first level of the car park because on this level the vehicles travel the longest distance in search of a parking space. Although the engines are already hot in scenario S1, the total emissions are the highest because the number of vehicles is about 33% higher than in S2 and S3. Analysis of the graph highlights a direct correlation between the number of vehicles moving and the hourly volume of CO_2_ emissions.

When moving on ramps, the CO_2_ emission values in scenarios S2 and S3 in which a combined uphill–downhill flow is recorded do not generate significant emissions compared to S1, although the transit is 33% higher because vehicles leaving the parking lot move on the ramp with engine braking, therefore with minimal emissions. Additional emissions are recorded only in the stop sector at the barrier. Difference in CO_2_ emission values between scenarios S2 and S3 is due to the over-fueling of cold engines in order to reach the regime for operation.

Figure 24 graphically presents the results of the hourly maximum values of NMVOC emissions for all representative areas related to the three scenarios (S1–S3).

In scenario S1, although the vehicles travel a significant distance inside the car park and the car park is filled to capacity (180 cars), emissions are lower due to the movement with engines that are already hot and due to the consideration of an average operating temperature of 25 °C. The major difference in NMVOC emissions generated in scenarios S2 and S3 compared to S1 appears as the influence of the factors:

- Starting vehicles with cold motors that generate emissions of NMVOC (unburned fuel);
- Vaporization of fuels from the tanks of parked vehicles.

Since the maximum NMVOC emissions are recorded in Scenario S2, it can be concluded that the critical factor of these emissions is the cold engine start and traveling.

When traveling on ramps in all three scenarios, the estimated values of NMVOC emissions are reduced because:

- Emissions from engine start-up on the ramp does not exist;
- The time spent by vehicles on the ramp is short, which makes evaporative emissions considerably negligible;
- The distance traveled by vehicles is small compared to the traffic on the flat area relating to the ground floor and two of the other floors.

Figure 25 presents the results of hourly NOx emission values for all representative zones related to the three scenarios (S1–S3).

In scenario S1, although vehicles travel a significant distance inside the car park and it is filled to capacity (180 cars), emissions are minimal due to the vehicles driving with engines that are already hot. The major difference occurs in scenario S2, where the starting of the vehicles leaving the parking lot becomes the critical factor. The trend is also maintained in the S3 scenario but with much lower NOx emission values. Thus, in the winter scenario S2 (0 °C) maximum hourly NOx emissions (approx. 29.14 g/h) are generated due to cold engine starts and operation of vehicles (rich fuel mixture and cold catalysts/catalytic reduction systems). Similarly, the summer scenario S3 (20 °C) maintains the dynamics of S2, but emissions decrease to 12.74 g/h due to the higher ambient temperature. The low values recorded on the ramps in all three scenarios demonstrate the easy vehicular circulation between levels does not generate significant NOx emissions because the cars leaving the car park drive on the ramp with their engine braking, therefore resulting in minimal emissions. However, the impact of the factors for driving on the ramp (f_r_) is observed. Analysis of the graph highlights a direct correlation between the thermal regime of the starting of the engines and the volume of NOx emissions.

Figure 26 shows the hourly values of NH_3_ emissions for all representative zones related to the three scenarios (S1–S3).

It is noted that the maximum NH_3_ emissions (approx. 0.42 g/h) are in the winter scenario S2 due to start-up and operation of vehicles with cold engines (incomplete chemical reactions due to cold catalysts, partial reduction of NOx to ammonia in the case of three-way catalysts during rich mixture operation specific to engine start-up, in the case of engines equipped with SCR during AdBlue dosing in the cold catalysts). In scenario S3, the beneficial influence of the external temperature on NH_3_ emissions is observed. The difference between scenarios S3 and S1 on the ground floor and the first floor is due to the different numbers of vehicles traveling through the MB-UPT car park. Regarding the driving of vehicles on the ramps and on the second floor, the influence of cold engines related to vehicles leaving the car park is observed.

Analysis of the graph highlights a direct correlation between the thermal regime of engine at start-up in circulation mode and NH_3_ emission concentrations.

The study will continue with a validation process based on experimental measurements of the concentrations of pollutants inside the MB-UPT car park (after the investment is completed). During the validation of the calculation methodology, hourly emissions calculations will be reperformed depending on the real structure of the motor vehicle fleet, the degree of loading, weather conditions, etc.

## 4. Conclusions

The need for this study derives from the need to build new, compact and multistory parking spaces in congested areas of large cities to streamline road traffic by freeing up lanes used for street parking spaces. On the other hand, for this type of buildings, environmental impact studies that also include a pollutant dispersion study are increasingly requested, respectively conclusions regarding the level of immissions in the neighboring built-up area.

Carrying out a dispersion study with the smallest possible margin of error requires the use of input data that are as close as possible to those expected to be encountered in the operation of car-park-type buildings. The most important data that must be estimated with high accuracy refers to the emissions resulting from different vehicle operating conditions. For the case study, these conditions refer to cold and hot engine operations, horizontal and ramp travels, cold start, fuel vaporization from the tanks of parked vehicles and the mode of operation of the car park (school activity period, weekend, vacation, sports/cultural events).

The article presents emission factors specific to various vehicle categories found in the specialized literature but also highlights that they require corrections to describe the operating regimes of both vehicles and car park operation modes. The methodology for calculating emissions specific to multistory car parks developed by the authors is based on the logical diagram of interactions between functional blocks made with elements specific to car parks and is treated as a case study for the aboveground MB-UPT car park.

The equations were developed by the authors based on an algorithm that includes corrected emission factors. The relationships were applied to calculate gas emissions in the MB-UPT car park, resulting in the present case study. In the article, based on the available data on the structure of the car park in Timiș County and depending on the number of spaces available in the car park, a structure of the car fleet similar to the one that would use the car park was anticipated.

The functionality of the car park was analyzed from the point of view of the hourly loading grades, specific to the operating mode of the MB-UPT car park (school activity period, weekend with/without sports/cultural events (occasionally), holiday in accordance with the UPT calendar).

For each of these periods, three scenarios correlating with the structure of the UPT academic year were analyzed and identified, for which hourly gas emissions inside the car park were calculated, with significant hourly intervals. The obtained results were presented in a table, and the spatial distribution of emissions was graphically drawn for CO emissions because this pollutant is the predominant pollutant in the case of car parks, but also because depending on the concentration of this pollutant the operation of the ventilation system will be adapted. From the analysis of the results, for each pollutant species analyzed, the relevant critical factor was determined in the context of the three scenarios.

The comparative graphs for each pollutant in the three scenarios show that:

- The analysis of the CO and NOx emissions graph highlights a direct correlation between the thermal regime of engine start-up and their quantity;
- The analysis of the CO_2_ emissions graph highlights a direct correlation between the number of vehicles traveling and their quantity;
- The analysis of the maximum NMVOC emissions graph highlights its significant increase in Scenario S2, thus resulting in the critical factor of these emissions being the cold engine start-up and operation and not the evaporation of fuels from the tanks of parked vehicles during the summer;
- The analysis of the NH_3_ emissions graph highlights a direct correlation between the thermal regime of engine start-up, the traveling of vehicles and the concentration of these emissions.

After the investment is completed, the authors aim to validate the method and the results under real operating conditions through experimental measurements. In the event that attempts to further validate the results through measurements of emissions, immissions, local weather data, or through modeling of the dispersion of noxious gases inside the parking lot reveal discrepancies between the local emissions estimated in the case study and measured/simulated values, the results will be analyzed additionally to reduce the estimated results’ uncertainty.

## Figures and Tables

**Table 1:** Emission factors (REF_eh_vc_) for vehicles by categories and European emission standards.

Motor	Fuel_cons_ [g/km]	CO [g/km]	CO_2_ [g/km]	NMVOC [g/km]	NOx [g/km]	NH_3_ [g/km]
**Gasoline**						
Non-Euro	77	7.178	239.514	1.936	1.235	0.002
Euro 1	66	4.962	205.034	0.992	0.454	0.088
Euro 2	66	2.998	199.754	0.528	0.247	0.107
Euro 3	66	2.827	208.623	0.300	0.105	0.032
Euro 4	66	0.954	214.942	0.187	0.064	0.031
Euro 5	66	0.946	214.931	0.163	0.047	0.014
Euro 6	66	0.397	214.894	0.052	0.032	0.013
**Diesel**						
Non-Euro	63	0.667	194.469	0.180	0.567	0.001
Euro 1	55	0.415	171.494	0.057	0.697	0.001
Euro 2	55	0.295	178.119	0.040	0.729	0.001
Euro 3	55	0.093	168.739	0.024	0.786	0.001
Euro 4	55	0.092	168.739	0.017	0.599	0.001
Euro 5	55	0.035	168.726	0.001	0.562	0.002
Euro 6	55	0.052	168.693	0.001	0.507	0.007
**Hybrid gasoline**						
Euro 4	34	0.649	134.313	0.164	0.037	0.031
Euro 5	34	0.649	134.302	0.156	0.037	0.031
Euro 6	34	0.075	134.275	0.051	0.014	0.031
**Plug-in hybrid electric vehicle (gasoline)**						
Euro 6	34	0.056	126.631	0.080	0.010	0.023
**Plug-in hybrid electric vehicle (diesel)**						
Euro 6	73	0.039	140.152	0.001	0.381	0.005

**Table 2:** Evaporative emission factors (EFVOCs) for motor vehicles in typical summer and winter conditions [22].

Vehicle Type: Gasoline PC		
Hourly Temperature [°C]	Equivalent Vapor Pressure [kPa]	NMVOC Pollutants [g/Vehicle/h]
−5	90	0.12
0	90	0.16
5	90	0.20
10	90	0.24
15	90	0.33
20	60	0.44
25	60	0.56
30	60	0.68
35	60	0.80

**Table 3:** Correction factors for driving on ramp (f_r_) [29].

Correction Factors/Pollutants	Ramp	
	Downhill (−10%)	Uphill (10%)
CO	0.76	1.66
NOx	0.69	1.59
NMVOC	0.86	1.32

**Table 4:** Temperature-dependent cold start emission factors for motor vehicles (f_cs_) in g/motor vehicle_start_ [29].

Emission Factors/Pollutants [g/Motor Vehicle_start_]	Temperature [°C]				
	−10	0	10	20	25
CO	9.78	6.36	4.31	2.26	2.07 *
NOx	0.83	0.51	0.20	0.1	0.09 *
NMVOC	1.74	1.11	0.75	0.40	0.36 *

* Note: The values presented were obtained by the authors using exponential extrapolation with the exponential regression method. Using this method for the extrapolation of 5 degrees above the value of 20 °C, the statistical uncertainty is in the range of ±5% to ±15% which represents a medium-low margin of error in the assessment of gaseous emissions.

**Table 5:** Cold start emission factor as a function of parking duration (f_ds_) [29].

Correction Factors/Pollutants [-]	Parking Duration [h]				
	<1 h	<2 h	<4 h	<5 h	>5 h
CO	0.18	0.46	0.58	0.67	1.00
NOx	0.45	0.89	0.97	1.00	1.00
NMVOC	0.18	0.46	0.58	0.67	1.00

**Table 6:** Correction factor for distance driven with a cold engine in car park (f_d_) [29].

Pollutant	Correction Factor for Distance Driving with a Cold Engine f_d_ [-]
CO	0.8
NOx	0.48
NMVOC	0.85

**Table 7:** Correction factor depending on the age of the motor vehicle fleet (f_fa_) [29].

Pollutant/Years Range	2020	2025	2030
CO	1	0.931	0.917
NOx	1	1.061	1.073
Benzol/HC	1	0.965	0.959

**Table 8:** The hourly emissions for CO, CO_2_, NMVOC, NOx, and NH_3_ for each representative area (S1).

	CO	CO_2_	NMVOC	NOx	NH_3_
	[g/h]	[g/h]	[g/h]	[g/h]	[g/h]
Ground floor	13.93	2903.54	16.75	5.97	0.29
Ramp GF_FF1	11.18	2329.03	1.50	4.58	0.14
First floor	16.64	3468.72	17.20	7.13	0.35
Ramp FF1_SF2	5.59	1164.52	0.75	2.29	0.07
Second floor	6.06	1262.33	15.42	2.59	0.13

**Table 9:** The hourly emissions for CO, CO_2_, NMVOC, NOx, and NH_3_ for each representative area (S2).

	CO	CO_2_	NMVOC	NOx	NH_3_
	[g/h]	[g/h]	[g/h]	[g/h]	[g/h]
Ground floor	266.67	2548.11	58.62	27.41	0.42
Ramp GF_FF1	14.04	2592.80	1.95	5.00	0.34
First floor	269.42	3101.41	59.88	29.14	0.52
Ramp FF1_SF2	7.02	1296.40	0.98	2.50	0.17
Second floor	262.45	1645.20	58.87	26.26	0.27

**Table 10:** The hourly emissions for CO, CO_2_, NMVOC, NOx, and NH_3_ for each representative area (S3).

	CO	CO_2_	NMVOC	NOx	NH_3_
	[g/h]	[g/h]	[g/h]	[g/h]	[g/h]
Ground floor	102.67	2494.51	47.02	11.01	0.25
Ramp GF_FF1	14.04	2592.80	1.95	5.00	0.20
First floor	105.42	3047.81	48.28	12.74	0.30
Ramp FF1_SF2	7.02	1296.40	0.98	2.50	0.10
Second floor	98.45	1591.60	47.27	9.86	0.16

**Table 11:** The total hourly emissions for CO, CO_2_, NMVOC, NOx, and NH_3_ in the scenarios S1, S2 and S3 for all representative areas.

	CO	CO_2_	NMVOC	NOx	NH_3_
	[g/h]	[g/h]	[g/h]	[g/h]	[g/h]
Ground floor GF_S1	13.93	2903.54	16.75	5.97	0.29
Ground floor GF_S2	266.67	2548.11	58.62	27.41	0.42
Ground floor GF_S3	102.67	2494.51	47.02	11.01	0.25
Ramp GF_FF1_S1	11.18	2329.03	1.50	4.58	0.14
Ramp GF_FF1_S2	14.04	2592.80	1.95	5.00	0.34
Ramp GF_FF1_S3	14.04	2592.80	1.95	5.00	0.20
First floor FF1_S1	16.64	3468.72	17.20	7.13	0.35
First floor FF1_S2	269.42	3101.41	59.88	29.14	0.52
First floor FF1_S3	105.42	3047.81	48.28	12.74	0.30
Ramp FF1_SF2_S1	5.59	1164.52	0.75	2.29	0.07
Ramp FF1_SF2_S2	7.02	1296.40	0.98	2.50	0.17
Ramp FF1_SF2_S3	7.02	1296.40	0.98	2.50	0.10
Second floor SF2_S1	6.06	1262.33	15.42	2.59	0.13
Second floor SF2_S2	262.45	1645.20	58.87	26.26	0.27
Second floor SF2_S3	98.45	1591.60	47.27	9.86	0.16

## Data Availability

The data presented in this study are available on request from the corresponding author.

## Abbreviations

The following abbreviations are used in this manuscript:

MB-UPT	Multistory building in University Politehnica Timisoara
UPT	University Politehnica Timisoara
EEA	European Environment Agency
NMVOC	Non-methane volatile organic compound
CO	Carbon monoxide
CO_2_	Carbon dioxide
NOx	Nitrogen oxides
NH_3_	Ammonia
INCERC	National Institute for Research and Development in Construction and Urbanism and Sustainable Territorial Development
WUT	West University of Timisoara
G	Gasoline
D	Diesel
HG	Hybrid gasoline
HEVG	Plug-in hybrid electric vehicle (gasoline)
HEVD	Plug-in hybrid electric vehicle (diesel)
GF	Ground floor
FF	First floor
SF	Second floor
